# Modeling the evolution space of breakage fusion bridge cycles with a stochastic folding process

**DOI:** 10.1007/s00285-015-0875-2

**Published:** 2015-04-02

**Authors:** C. D. Greenman, S. L. Cooke, J. Marshall, M. R. Stratton, P. J. Campbell

**Affiliations:** 1 School of Computing Sciences, University of East Anglia, Norwich, UK; 2 The Genome Analysis Centre, Norwich Research Park, Norwich, UK; 3 Cancer Genome Project, Wellcome Trust Sanger Institute, Hinxton, UK; 4 Department of Haematology, University of Cambridge, Cambridge, UK

**Keywords:** 05A05, 60G99, 92B05, 92D15, 92D20

## Abstract

Breakage–fusion–bridge cycles in cancer arise when a broken segment of DNA is duplicated and an end from each copy joined together. This structure then ‘unfolds’ into a new piece of palindromic DNA. This is one mechanism responsible for the localised amplicons observed in cancer genome data. Here we study the evolution space of breakage–fusion–bridge structures in detail. We firstly consider discrete representations of this space with 2-d trees to demonstrate that there are $2^{\frac{n(n-1)}{2}}$ qualitatively distinct evolutions involving $n$ breakage–fusion–bridge cycles. Secondly we consider the stochastic nature of the process to show these evolutions are not equally likely, and also describe how amplicons become localized. Finally we highlight these methods by inferring the evolution of breakage–fusion–bridge cycles with data from primary tissue cancer samples.

## Introduction

**Fig. 1:**
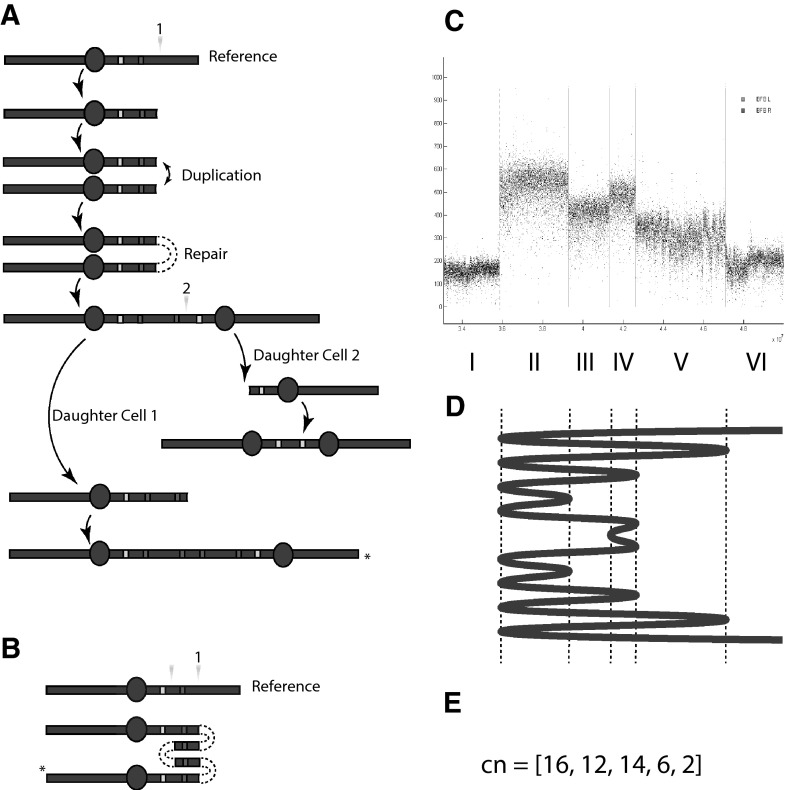
The BFB process. In **a** we have a representation of a chromosome, the *circle* being a centromere, the *red* and *yellow* markers hypothetical genes duplicated and deleted throughout this process. A DNA break (at position of *orange triangles*) followed by duplication and repair results in a palindromic chromosome with two centromeres. Spindles grab each centromere and contract during cell division resulting in another break and the cycle continues. In **b** the BFB product (*asterisk*) is folded relative to the original reference genome. In **c** is an amplicon formed through a BFB process. The *horizontal axis* is genomic position, the *vertical axis* is read depth. Vertical lines indicated detected BFB folds. In **d** is the predicted BFB folding pattern. In **e** is the copy number profile, $cn$ (color figure online)

Breakage–fusion–bridge (BFB) cycles potentially arise whenever a stretch of DNA is broken and a cell division cycle takes place. The first stage in this division process is DNA replication, where duplication will take place up to the DNA break, leaving two exposed ends. Prior to cell division, the cells checkpoint machinery will look for mistakes and the two exposed ends may be erroneously joined together by double stranded break repair. This results in a palindromic sequence of DNA, often containing a duplicated centromere (see Fig. 1a). Spindles then attach to centromeres, which then contract during cell division to pull a chromosome into each daughter cell. However, if each centromere of this dicentric chromosome is to successfully relocate to distinct daughter cells, the DNA between the centromeres has to snap, and so each daughter cell will have a centromere on a DNA segment with an exposed (broken) end. This process of duplication, end repair and DNA breaking can then continue with each cell division and the process repeat itself in a cascade of BFB cycles.

Note that the first break in the cascade occurs on the longer arm of the chromosome to the right of the centromere, as presented in Fig. 1a (we shall refer to this as the q arm, and the other as the p arm). When the dicentric chromosome forms, the material between the two centromeres is entirely from the q arm, and we have two copies of the p arms at the extremities. The next break then occurs between the two centromeres and is thus again in the q arm. We then find that all breaks of a BFB cascade occur in the same arm of the chromosome.

The process is unlikely to continue indefinitely because eventually repair machinery will attach exposed ends to other portions of the genome to produce a translocation, for example, or telomerase may cap the end to produce a somatic telomere. However, this process of repeatedly duplicating, repairing and unfolding is known to produce complex rearrangements of the original genomic assembly, and are frequently observed in cancer genomes (Bignell et al. 2007), having originally been discovered in the genomes of zea mays plants (McClintock 1941). The complexities of these rearrangements have also been examined algebraically in Kinsella and Bafna (2012a, b), where algorithms to infer the breakage fusion bridge histories from observed copy number data were developed. These were improved to a linear time algorithm in Zakov et al. (2013), and to analyse noisy data in Zakov and Bafna (2014). In this work we consider a distinct combinatorial problem, investigating the space of possible structures that can arise from such a process.

These genomic rearrangements closely resemble paper folding operations in origami where paper is repeatedly folded in upward and downward directions. When the paper is unraveled, we obtain a series of troughs and peaks which can be represented as a binary sequence. These sequences can be recursively generated and serve as examples of automaton (Allouche and Shallit 2003), which gives us a starting point to represent BFB processes.

There are some key differences to note however. Whilst paper is intrinsically the same material at all positions, DNA is composed of a variable sequence of nucleotides and subsequently is identifiably unique along its length *prior* to the BFB duplication process (DNA repeats are ignored). We can thus label each point along the original DNA sequence with its genomic position and consider how these labels are duplicated in the BFB process. By comparing the positions on the BFB product with the original *reference* sequence, we can fold the BFB product so the same labels (i.e. reference positions) are vertically aligned, such as in Fig. 1b, where three folds are required. Note that these folds are located at precisely the two reference positions of DNA repair in the BFB cycles. The term *fold* and the folded structure relative to the reference will be used in the majority of the work. We will also use *breakpoint* to refer to the reference position of the fold. The stretch of DNA between two consecutive folds will be referred to as a *segment*.

This representation mirrors that observed experimentally. In Fig. 1c, for example, we have a sample of next generation sequencing from a primary breast cancer sample (PD4875) used in ICGC (2010). The horizontal axis represents the reference position along chromosome 11, and the vertical axis the experimental signal (sequence read depth; Pleasance et al. 2010a, b). The green and red vertical lines indicating positions where paired reads were identified that were consistent with BFB folds pointing left and right, respectively. These positions segment the reference genome into six regions $I,II,\dots ,VI$. We see that the signal is relatively constant within each of these regions. Note also that all these positions (within 36–50 Mb) lie in the same arm of the chromosome, downstream of the centromere at position 53.7 Mb. Collectively, these results are indicative of a sequence of BFB cycles, and we will later infer the likely underlying folding structure, the prediction indicated in Fig. 1d.

This inference relies in part on the linear relationship between the experimental signal and the number of copies of DNA ‘folded’ across a given reference region, the *copy number profile*, summarized in Fig. 1e. For this prediction, we see that region $II$ (with the highest signal) has a predicted copy number of $16$; each of eight folds on the left side of the region accounting for two genomic copies.

These data constitute an example of an ‘amplicon’, which are frequently observed in cancer genome data. These are clusters of rearrangements with a high signal in the reference genome, indicating an abnormally large number of copies are present in the cancer genome. These ‘amplified’ regions are usually restricted to a few megabases of DNA, a small portion of a typical human chromosome. The BFB process is one mechanism by which these events can arise (Bignell et al. 2007; McClintock 1941). Next generation sequencing technologies mean we can now visualize these events in great detail, producing extensive catalogs of the mutations involved (Pleasance et al. 2010a, b), from which the etiology of these events can then be investigated (Greenman et al. 2011; Raphael et al. 2003).

In this work we consider several interesting questions that naturally arise from BFB processes. Firstly, consider the problem of how best to represent this process. It is discrete, both in terms of the type of folded structures that can arise, and in terms of the reference nucleotide positions of the folds. By introducing a discrete representation of the BFB process, we provide a coherent representation of the genomic conformations that can arise in BFB ‘space’. Furthermore, this structure allows us to measure the size of this space, proving that there are $2^{\frac{n(n-1)}{2}}$ qualitatively distinct evolutions given $n$ BFB cycles. We also approximate the reference nucleotide positions of the folds as a continuous stochastic process. This allow us to explore the probability of occurrence for each of the $2^{\frac{n(n-1)}{2}}$ elements of this space. Furthermore, this provides some understanding into why amplicons are so localised in the genome.

In these analyses we have to make some assumptions regarding the nature of breakpoints, in particular whether breakpoints from previous BFB cycles are implicated in subsequent cycles. There is some debate in the literature over the terminology and nature of breakpoint reuse by rearrangements (Sankoff 2009). Breakpoint reuse can refer to the reuse of a specific region or to the reuse of an exact position, for example. A cross species comparison (Lemaitre et al. 2009) has shown breakpoint positions correlate with transcription and chromatin conformation. It is also well established that there are fragile regions of the genome prone to double stranded breaks (Bignell et al. 2010). This may lead to some clustering of breakpoints, which are unlikely to be uniformly distributed along the chromosome, although it is unclear how these observations apply specifically to the BFB process.

This suggests that reuse of breakpoint regions is quite plausible. However, detailed copy number analyses of hundreds of cancer samples across these regions (Bignell et al. 2010) reveal that breakpoint positions are highly variable within each fragile site, suggesting that two breaks occurring at precisely the same position is unlikely and the reuse of a specific breakpoint position will not be common. Furthermore, if BFB breaks do occur at the same position, we should find left and right facing folds occurring at the same position in a reasonable proportion of cases. By sequencing reads that bridge rearrangements it is possible to obtain sequence down to the single nucleotide level and search for these events (Bignell et al. 2007; Campbell et al. 2008). These studies reveal the presence of small shards of inserted DNA, and micro-homologies arising during the repair process, but evidence of multiple rearrangements arising within the scale of a single paired read is hard to find.

Thus although some clustering suggest breakpoint regions maybe reused, the exact breakpoint positions are unlikely to be reused. We thus assume that each breakpoint is implicated by a single rearrangement. Although proximal rearrangements will likely occur, the resolution of modern sequencing means we can resolve many of these cases, and our assumption is reasonable.

We also assume that the distribution of breakpoints is uniform when we analyze the stochastic properties of the process. This is likely to be approximate, but is the logical model to explore until we have a better understanding of the distribution of breakpoints from BFB processes.

## Overview of approach

We now present an overview of the approach and outline each section in the paper.

**Fig. 2:**
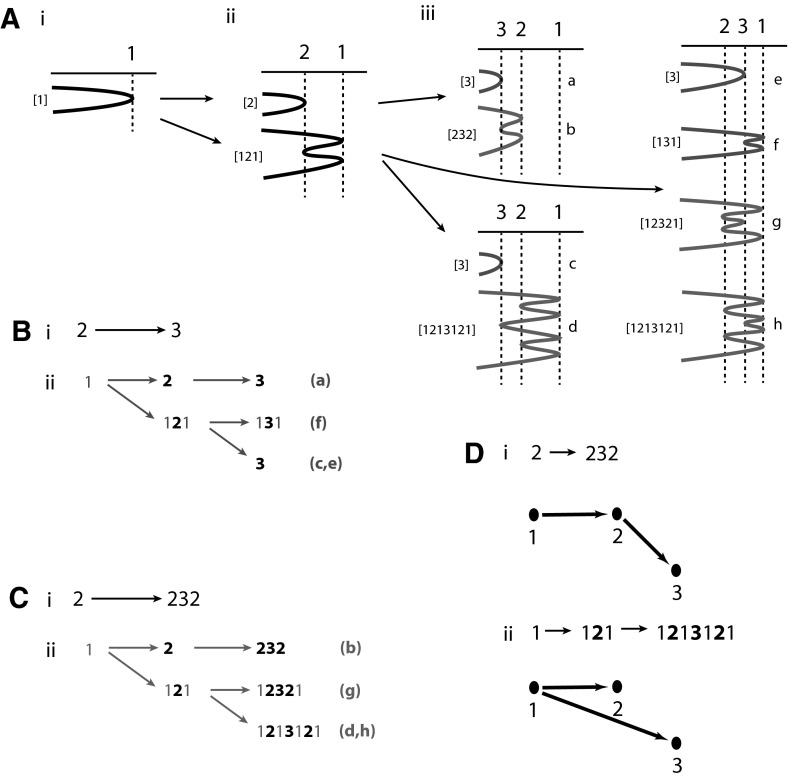
Counting BFB structures. In **a**(*i*) we see one structure with one fold, in **a**(*ii*) two structures with two folds, and in **a**(*iii*) $2^{\frac{3(3-1)}{2}}$ structures arising from three folds, labeled a, b, ..., h. The fold word for each structure is given in square brackets. In **b**, **c**(*i*) we see the two evolutions involving two fold number $2$ and $3$; $2 \rightarrow 3$ and $2 \rightarrow 232$. In **b**, **c**(*ii*) the corresponding $2^2$ induced evolutions on three folds $1$, $2$ and $3$ are provided. In **d**(*i*) we have a poset tree for evolution $\mathbf{2} \rightarrow \mathbf{232}$, and in **d**(*ii*) an induced poset tree for evolution $1 \rightarrow 1\mathbf{2}1 \rightarrow 1\mathbf{2}1\mathbf{3}1\mathbf{2}1$

In Fig. 2 we see the range of structures that can arise from three BFB cycles. The initial cycle gives rise to the structure in Fig. 2a(i) with a single fold located at reference position $1$. This structure has two segments in Fig. 2a(i) (either side of the fold) that can contain the breakpoint from the next BFB cycle and form a second fold (at reference position $2$). This results in two possible structures, as given in Fig. 2a(ii). These two structures have, respectively, two and six segments that a new fold can form along, giving the eight possible structures portrayed in Fig. 2a(iii). Note that we have $1$, $2$ and $8$ structures after $1$, $2$ and $3$ BFB cycles. Generalizing this observation suggests that $n$ BFB cycles results in $2^\frac{n(n-1)}{2}$ possible structures. We would like to understand this pattern.

Note that $2^\frac{n(n-1)}{2}=2^{1+2+3+\dots +n-1}=\prod _{i=1}^{n-1}2^i$. It is thus desirable to associate $2^i$ new structures with any structure arising from $i$ cycles of a BFB. However, the two structures from two BFB cycles in Fig. 2a(ii) led to two $(a,b)$ and six $(c,d,e,f,g,h)$ new structures, respectively, rather than the $2^2$ we would like, so we are searching for a deeper connection. This requires the introduction of some machinery.

Notice that if we walk along any BFB structure we can read off the labels of the folds. For example, starting from either end of structure $d$ of Fig. 2a(iii) and reporting $1$, $2$ or $3$ whenever we change direction at a fold located at the corresponding reference position, results in the word $1213121$. These *fold words* are given in square brackets for each structure in Fig. 2a. We can also write the evolution for $d$ in terms of the such words, where we have $1 \rightarrow 121 \rightarrow 1213121$. This raises two immediate problems. Firstly, we need to determine the nature of possible words that may arise from such a process. Secondly, if we are given a word that represents the final product, can we undo the word, reverse engineer the process, and infer the evolution that has taken place. This is explored in more detail in Sect. 3.

Notice in Fig. 2a(iii) that we actually have two structures ($d$ and $h$) associated with the word $1213121$. To understand the size of BFB space we then have three problems; firstly, determine how many different structures there are for each word, secondly, determine how many different words there are given $n$ BFB cycles, and thirdly, establish that $2^\frac{n(n-1)}{2}$ counts the size of the entire space.

In Sect. 4 we consider the first question. The two structures for $1213121$ arise because the third fold $3$ can have a reference position either to the left or to the right of the reference position for fold $2$. If $x_1,x_2,x_3$ are the reference positions for folds $1$, $2$ and $3$, we have choices $x_3<x_2<x_1$ or $x_2<x_3<x_1$. Such conditions are known as linear extensions that arise from a partially ordered set (*poset*) (Neggers and Kim 1998). These order relationships can be represented by the rooted, directed tree in Fig. 2d(ii). Here the nodes $2$ and $3$ are located on distinct branches. This represents the fact that no order relation exists between the two corresponding reference positions. Conversely, root node $1$ is located on branches common to $2$ and $3$. This reflects the fact that $x_1$ has order relations with both positions $x_2$ and $x_3$. In Sect. 4 we explore how to construct these poset trees for any fold word, and how this allows us to count the number of associated BFB structures. This involves the construction of more general objects known as 2d-trees in Sect. 4, that will ultimately help us demonstrate that the size of BFB space on $n$ cycles is $2^\frac{n(n-1)}{2}$.

In Sect. 5 we derive this expression. This requires an association of $2^2$ new structures to both elements of Fig. 2a(ii), rather than the two $(a,b)$ and six $(c,d,e,f,g,h)$, as mentioned above. Curiously, if we reintroduce a first event, we get the desired pattern. The two possible evolutions [Fig. 2a(ii)] involving two initial folds $1$ and $2$ are $1 \rightarrow 2$ and $1 \rightarrow 121$. If these become the second and third events, the evolutions become $2 \rightarrow 3$ and $2 \rightarrow 232$, respectively. In Fig. 2b(ii), c(ii) we then see the possible evolutions that include a first fold $1$ such that evolutions on folds $2$ and $3$ are preserved (in bold). Note in particular that both cases result in three different word evolutions with $2^2$ distinct structures; $2 \rightarrow 3$ is associated with $a,c,e,f$ (in green) and $2 \rightarrow 232$ is associated with $b,d,g,h$ (in red). This is precisely the pattern we require. When this is translated into the language of poset graphs we find that the poset graphs are related. For example, in Fig. 2d(ii) we find that the poset graph for the induced evolution $1 \rightarrow 121 \rightarrow 1213121$ can be obtained from the poset graph for evolution $2 \rightarrow 232$, by moving the edge bridging nodes $2$ and $3$ to an edge bridging nodes $1$ and $3$. These tree operations, their relationship to fold words and the size of BFB space are explored in Sect. 5.

This description of the BFB process has thus far been discrete in nature. However, the fold positions can occur at any nucleotide along the chromosome and we are left to wonder if these discrete structure arise with equal probability. The simplest approach to this is to assume that the break occurs along the length of the structure with uniform probability. This is likely to be approximate, as discussed above, but should give us some idea of behavior and a sense of how the probability mass is distributed amongst the $2^\frac{n(n-1)}{2}$ possible structures. By deriving length distributions for these structures we explore these issues in Sect. 6 and show that these structures arise with probabilities that are far from equal.

This will provide a reasonable understanding of BFB space. We would like to know how this understanding can help us interpret real data. In particular, a method to infer the historic BFB process given some experimental observations is desirable. Paired end sequencing provides examples of BFBs such as the primary breast data in Fig. 1c. In Sect. 7 we use the machinery to help identify the underlying structures from data, noting two things. Firstly, although the number of possible BFBs is vast, the number of likely structures can be very small. Secondly, degeneracy of the space means that different evolutions can have identical copy number profiles. This can make identification of a single explanatory evolution very difficult.

Concluding remarks complete the paper. The majority of proofs have been consigned to the Appendix.

## Word representations of BFB processes

We now consider the word representation of the BFB process in more detail, both in a forward direction, reflecting the evolution of the BFB process, and backward, indicating how to unravel a BFB folded structure, reverse the process, and infer the events that have taken place.

### The forward process

The BFB process can be described as an iteration scheme on a word of symbols. This follows ideas from paper folding sequences, where the binary letters of words represent peaks and troughs that run along an unfolded piece of paper formed from a series of folding operations. These words can be constructed by an iteration of word operators each of which depend upon whether the folding action was up or down (Allouche and Shallit 2003).

For BFBs we have to generalize this somewhat. Firstly, binary sequences prove inadequate, so we introduce a symbolic ‘language’ to draw from, in terms of the (reference) positions of folds. This representation is different to that in Kinsella and Bafna (2012a), where a language in terms of the regions of the reference genome that bridge these positions was used to investigate patterns of copy number arising from BFB processes.

**Fig. 3:**
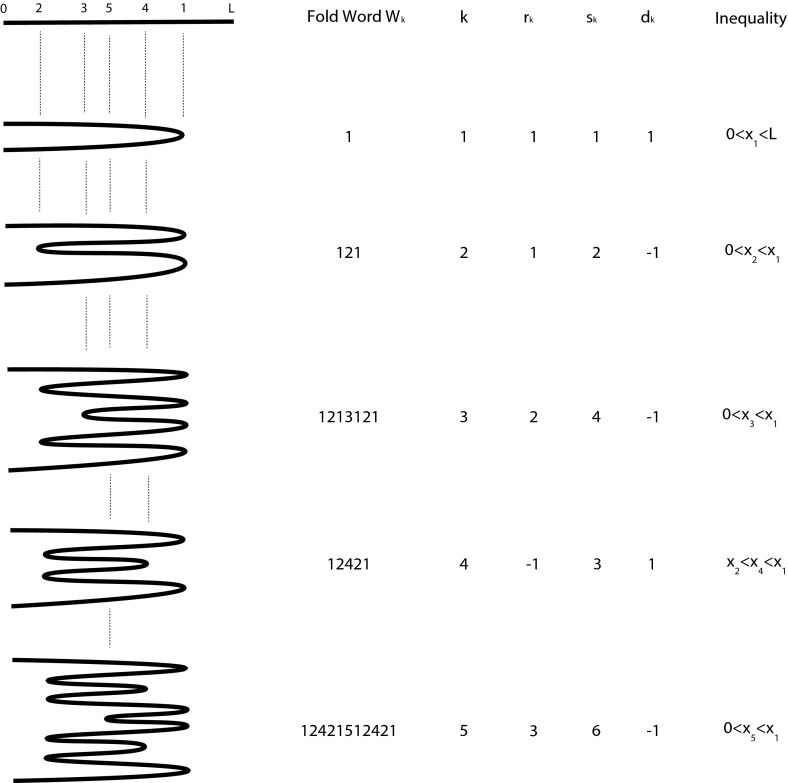
The *first column* indicates a sequence of five BFBs operating on interval $[0,L]$. The *second column* gives the fold word. The *third column* indexes the BFB. The *fourth column* indicates the fold sequence. The *fifth* and *sixth columns* are the cumulative sequence and the directional signature. The *final column* provides inequalities satisfied by each fold position $x_i$ of fold $i$

Consider the example in Fig. 3. Here a segment of DNA, represented by interval $[0,L]$, undergoes a series of five BFB cycles (first column). This results in five folds positions which partition the reference genome into six regions.

We now implement the BFB process. Firstly, we have a break in our segment at the first fold, labeled $1$, position $x_1$. We suppose the DNA to the left of the break is duplicated and the right side is lost to a different daughter cell. The two duplicated ends at position $x_1$ are then stitched together to form one new structure. If we traverse the structure from one end to the other, reporting fold numbers when we reach a fold; this gives the simple *one-fold* word $W_1=1$.

We continue the process, the next fold occurring at position $x_2$, this time the fold pointing to the left. The resulting structure has three folds. Walking through the structure reporting folds we obtain the *two-fold* word $W_2=121$.

Note also that the word is palindromic, if we reverse the order of the symbols we obtain the same word. This reflects the chromosome symmetry; if we turn the chromosome upside down, we end up with an identical structure.

Note that we had two choices to construct the fold at reference position $x_2$. Considering the second structure formed, we can either break at the upper copy of $x_2$ and duplicate the DNA below, or break at the lower copy and duplicate the DNA above, the same product results. This symmetry is true in general, which we summarize.

#### *Remark 3.1*

If a BFB fold is positioned a length $l$ from one end of a BFB product, with the upper portion duplicated, the BFB product cannot be distinguished from that arising when a BFB fold positioned a length $l$ from the other end with the lower portion duplicated.

The palindromic nature of BFB products then means we always have two choices to place the fold position for any given product. In what follows, we always assume that we are duplicating DNA above the position of the fold, with respect to the representations drawn in Fig. 3. Note that this only applies to a palindromic product and so does not apply to the very first BFB event, for which every fold position and duplication gives a unique product.

We now continue the process, iteratively building up the word. The next new fold is at position $x_3$, occurring after the third fold of the third product. We thus keep the word containing the first three folds $121$, insert the new fold $3$, and add the first three folds in reverse order $121$, resulting in *three-fold* word $W_3=1213121$. This fold is then deleted by the fourth BFB; the corresponding fold is positioned at reference position $x_4$, occurring immediately after the second fold in the structure and we obtain *four-fold* word $W_4=12421$, losing symbol $3$. Although five folds take place, the final conformation $W_5=12421512421$ then only contains four fold numbers $1,2,4$ and $5$. This can happen generally, if the fold occurs in the upper half we lose the middle position, which must be the previous BFB location, and information is lost. Furthermore, if we simply implement the BFBs $1,2,4$ and $5$ we get the same product.

Note also that in Fig. 3 the first BFB involved the loss of the segment to the right of $x_1$, resulting in a fold pointing to the right (relative to the reference). All copies of DNA that were originally to the right of this fold have been permanently lost. As noted in the introduction, all the reference positions of folds from a BFB cascade occur on one side of the centromere. To simplify exposition, we always assume that the fold positions occur on the ride side of the centromere. The ends of the BFB structure then extend in a leftward direction (towards the centromere). For any structure with fold reference positions occurring on the left side of the centromere, we can just reverse the direction of the reference.

We summarize the word representations of a BFB process as follows.

**Word construction**

We initialize with $W_1=1$ and use recursion $W_i=W_{i-1}(b_i)i \overline{W_{i-1}(b_i)}$ where $b_i$ is the number of duplicated folds, $W(b)$ is the subword of $W$ containing the first $b$ symbols, and $\overline{W}$ is the word $W$ with symbols in reversed order.

### The reverse process

We now consider the opposite problem; given a BFB word, we need to reverse the process and identify the events that have taken place. This represents the typical inference problem in genomics, where we have the final structure of a genome and wish to reverse engineer the process to identify the evolutionary history. This can be achieved by identifying the unique BFB fold that demarcates the centre of the palindromic structure and undoing the duplication. For example, the BFB sequence of Fig. 3 resulted in *five-fold* word $W=12421512421$. The center fold $5$ is undone, leaving palindrome $12421$. We then undo $4$ to leave $12$, which must derive from palindrome $121$. Undoing $2$ and then $1$ completes the sequence and the evolution of folds is $1,2,4$ and then $5$. Note that we have reconstructed a reduced evolution, not the full list; fold $3$, which was deleted by fold $4$, is not included. Note also that $1,2,4,5$ is precisely the order that the symbols first appear in the final word $W=12421512421$.

In general we have the following result.

#### **Theorem 3.1**

A fold word is a viable representation of a BFB process if and only if it can be reversed with the following algorithm. This identifies the unique minimal sequence of BFB cycles responsible for the word.

*STEP 1*: Take palindromic word $W=Xn\overline{X}$ and undo fold $n$ to leave $X$. Output $n$.
*STEP 2*: Identify the rightmost uniquely occurring symbol $m$ such that the fold word is $ZYm\overline{Y}$ for some (possibly empty) subsequences $Z$ and $Y$ which do not contain $m$. Undo $m$ and contract to word $W=ZY$. Output $m$.
*STEP 3*: If $W$ is empty the evolution is the reverse of the output, else repeat *STEP 2*.

For a viable BFB fold word $W$, the evolutionary order of BFBs is precisely the order that their corresponding fold number first appears in the word.

This is largely the same as the process described in Kinsella and Bafna (2012a), which reverse engineered a word representation based on regions rather than folds. Thus if the entire genomic sequence of a fully assembled chromosome arising from a set of BFB cycles is known, the process can be reversed and the unique minimal sequence of BFBs that lead to that sequence provided. Unfortunately, experimental data does not contain such detailed information. The copy number profile, for example, is a more typical experimental observable, indicating the number of times different regions are present, but not the order that they are present in the chromosomal structure. Furthermore, we have not considered the random nature of the process and in particular the different orders the fold positions can take. To help deal with these issues we next introduce a representation which captures the events that take place, rather than the sequence generated.

## BFB posets

**Fig. 4:**
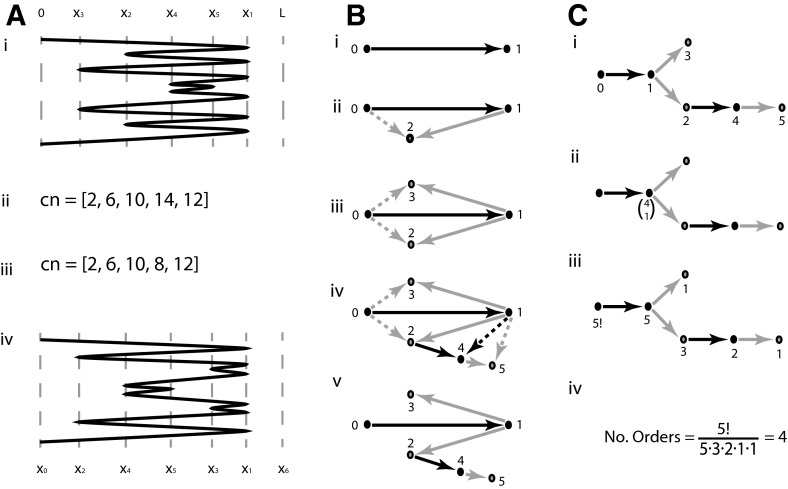
Poset of folding structures for a given BFB sequence. In **a**(*i*), (*iv*) we see two possible arrangements resulting from reduced representation $[1,1,2,2,1]$. a(ii), (iii) gives the copy number profiles of each. In **b** we see the poset graph construction representing the possible orders of positions. Nodes represent folds, edges represent inequalities between fold positions in the reference. *Solid* and *dashed edges* indicate *major* and *minor* edges, respectively. *Black* and *orange edges* indicate *plain* and *flipped* edges. Trees in **c** indicate how Theorem 4.2 is used to count the number of possible orders in the poset (color figure online)

We now have a word representation of the BFB process. This representation is not unique and there may be several qualitatively distinct structures for each BFB word. For example, the two structures in Fig. 4a(i), (iv) both correspond to word $1213145413121$, yet the two copy number profiles [Fig. 4a(ii), (iii)] are clearly distinct. In this section we count the number of structures for a given BFB word by constructing a suitable *partially order set* (poset). This is derived from a *2-d tree*, which is required for the more general problem of estimating the total number of evolutions in Sect. 5.

To do this, we first obtain a *fold sequence* representation for any given *n-fold* word. These sequences each contain $n$ integers and captures some nice properties of the structure, and offer a more analytically tractable formulation.

### Fold Sequences

Consider the evolution portrayed in Fig. 3. After each BFB we have a folded structure with a finite set of DNA segments going forward and backward between fold positions relative to the reference. We construct a fold sequence ${r_n}$ to represent these structures as follows. In order to specify a BFB cycle we have to indicate where the next fold is positioned on the current structure. The symmetry of the process (Remark 3.1) means we can specify that the duplication will always occur from one end, so we choose the top end of each structure as presented in Fig. 3. We then have to indicate which segment the next BFB fold will occur upon. For reasons described below, the segment immediately after the mid point is labeled $1$ and the labels of segments before or after are obtained by counting backward or forwards along the structure, respectively. The value $r_n$ is then the label for the segment containing the $n$th BFB fold.

Thus for the example of Fig. 3 we start with one segment. The first BFB occurs on this segment so we trivially write $r_1=1$. This produces two segments, and so two choices for the location of the next BFB fold. In our example this occurs on the edge below the midpoint, so we have $r_2=1$, producing four segments. The next BFB occurs on the last segment, two segments after the midpoint, so $r_3=2$. The next BFB loses the 3rd BFB fold, occurring two segments before the midpoint, so counting back from $1$, we have $r_4=-1$. The final BFB gives us $r_5=3$ so we have fold sequence $\mathbf{r}=[1,1,2,-1,3]$, as indicated in the fourth column of Fig. 3.

We noted previously that because the 3rd BFB is deleted by the 4th, the end product can be obtained by simply implementing the undeleted BFBs. Note that the 4th BFB fold can be positioned on the third segment after the midpoint of the structure arising from the 2nd BFB cycle. We can thus equally represent the final structure with *reduced fold sequence* $\hat{\mathbf{r}}=[1,1,\mathbf{1},3]$. Note that this can be derived from the fold sequence $\mathbf{r}=[1,1,2,-1,3]$ by absorbing the negative value $-1$ into the preceding value $2$, giving new (emboldened) value $1$.

Consider the cumulative sum of the fold sequence, the *cumulative sequence* $\mathbf{s}=[1,2,4,3,6]$. We have two observations. Firstly, $2s_n$ is equal to the number of segments of the structure after the $n$th BFB cycle. For example, in Fig. 3, the structure following the 3rd BFB has eight segments, and $2s_3=8$. Secondly, values $(-1)^{s_n+1}$ provide a *direction sequence*, $\mathbf{d}=[1,-1,-1,1,-1]$. Each number $d_n$ gives the direction of all copies of the $n$th BFB fold, relative to the reference. For example, all copies of the fold from the 2nd BFB, at position $x_2$, point to the left $(d_2=-1)$.

Representing the process this way thus enables us to capture some nice properties. We summarize this as follows.

#### **Theorem 4.1**

Any $m$-fold word representing a BFB process has an equivalent representation as a *fold sequence* of $m$ integers $\{r_n,n=1,2,\dots ,m\}$ that satisfies $-s_n < r_{n+1} \le s_n$ where $s_n=\sum _{i=1}^{n}r_i>0$ is the *cumulative sequence*. The number of segments of the structure after $n$ BFB cycles is $2s_n$. Each term $r_n$ in the sequence represents a BFB event. Negative terms indicate deletion of previous BFBs which shorten the structure. The *reduced fold sequence* contains strictly positive values and is obtained by combining $r_{n-1}$ and $r_n$ into the single term $r_{n-1}+r_n$ whenever $r_n \le 0$. This is repeated until only positive terms remain. The number of reductions using $r_n$ equals the number of BFB events deleted by the $n$th event. The direction of all copies of folds from the $n$th BFB is given by *direction sequence* $d_n=(-1)^{s_{n}+1}$.

We now have an algebraic representation of BFBs. We will see that each representation can account for many different BFB structures. The two structures given in Fig. 4a(i), (iv) are both represented by fold sequence $[1,1,2,2,1]$, for example. We build this example sequentially. We start with a single segment $[0,L]$ of length $L$ which undergoes a BFB at position $x_1$, where $x_0<x_1<L$ and $x_0=0$ is the reference position at the ends of the structure. This duplicates the segment $[0,x_1]$ and loses the right end from $x_1$ to $L$. We then have a structure with a single fold which we associate with word $W_1=1$. The next BFB fold occurs on the segment after the midpoint, represented as word $W_2=121$. The fold occurs at some position $x_2$ where $0<x_2<x_1$. The third fold then occurs two segments after the midpoint, where $r_3=2$ and $s_3=4$. This is represented by word $W_3=1213121=W_2(s_3-1)3\overline{W_2(s_3-1)}$. The third fold position $x_3$ occurs on the segment $[x_0,x_1]$, resulting in inequality $x_0<x_3<x_1$. This can be equivalently written as $x_{W_{2,s_3}}<x_3<x_{W_{2,s_3-1}}$, where subscript $W_{k,n}$ is the $n$th letter of $k$-*fold* word $W_k$ (if $W_k$ has $K$ symbols in total, we define $W_{k,0}=0=W_{k,K+1}$). We thus find that there are several order relationships on the reference positions of the BFB folds; we have a *partially ordered set* (poset).

The general situation is described in the following result.

#### **Lemma 4.1**

If $x_n$ is the reference position of the fold arising from the $n$th BFB cycle, and $d_n$ is the $n$th element in the corresponding direction sequence, then we find that the following partial orderings apply to the positions of the BFB folds:

$$\begin{aligned} \left\{ \begin{array}{l l l} d_n = -1 &{} \Rightarrow &{} x_{W_{n-1,s_n}}<x_n< x_{W_{n-1,s_n-1}}\\ d_n = 1 &{} \Rightarrow &{} x_{W_{n-1,s_n-1}}<x_n< x_{W_{n-1,s_n}}\\ \end{array} \right. \end{aligned}$$

For example, from fold sequence $[1,1,2,2,1]$ we obtain restrictions $0<x_1<L$, $0<x_2<x_1$, $0<x_3<x_1$, $x_2<x_4<x_1$ and $x_4<x_5<x_1$. There are several different orders that satisfy these criterion, Fig. 4a(i), (iv) being two such examples, where a(i) has order $0<x_3<x_2<x_4<x_5<x_1<L$ and a(iv) has order $0<x_2<x_4<x_5<x_3<x_1<L$. Note that this reordering of the fold positions does not change the number of copies of each fold, but can alter the copy number profiles [Fig. 4a(ii), (iii)].

### 2-d trees

It is natural to attempt to count and construct the different orders we get for a single fold sequence. We do this with the aid of a 2-d tree construct; a special kind of directed graph that generalizes the notion of a tree, as exemplified in Fig. 4b. This construction will be important when we count the total number of possible evolutions from $n$ BFB cycles in the next section.

When constructing a standard rooted tree, we can build from the root, recursively extending the tree with a single node and edge from a node that is already present. A 2-d tree differs in this respect; once we have one edge and two nodes, each new node has two *parent* nodes already present. Two edges are then constructed from these two nodes to the new node (Bentley 1975).

We construct a 2d-tree as follows. Each new node represents a BFB cycle, with label $n$ representing the $n$th fold. An edge represents an ordering relation. When the $n$th fold is formed, it is positioned on a segment between two pre-existing folds with positions $x_a$ and $x_b$, where we have $a<b$, without loss of generality. We then construct two directed edges from nodes $a$ and $b$ to $n$. For example, the second fold in Fig. 4a(i) has position $x_2$ with $x_0<x_2<x_1$, we thus construct two edges from nodes numbered $0$ and $1$ to a node labeled $2$ as given in Fig. 4b(ii).

We next require some classes for these edges.

#### **Definition 4.1**

Each pair of edges introduced during the 2d-tree construction consists of a *major* and *minor* edge. The *major* edge (represented as solid edges in figures), extends from the node with greater value $b$, and the *minor* edge (dashed), extends from the other node labeled $a$.

#### **Definition 4.2**

Each pair of major/minor edges and corresponding daughter node are either *plain* or *flipped*. If the word $W_{n-1}$ prior to the formation of fold $n$ changes from $XabY$ to $Xana\overline{X}$ (where $b>a$, and $X,Y$ are possibly empty subwords), the two edges and daughter node are *plain* (black). If they change from $XbaX$ to $Xbnb\overline{X}$, they are *flipped* (orange).

For example, in Fig. 4b(iv) we see node numbered $4$ extending from nodes $1$ and $2$. The major edge (solid) then extends from the larger source node numbered $2$. This node represents the introduction of the $4$th fold where word $1213\mathbf{12}1$ becomes $1213\mathbf{141}3121$. Because the two source nodes are increasing $\mathbf{12}$ in the word, both edges and daughter node are termed plain (black). Conversely, the $5$th fold arises when $12131\mathbf{41}3121$ becomes $12131\mathbf{454}13121$ so the edges and node are flipped (orange).

The following observation is important when we later consider the number of possible evolutions from a fixed number of BFB cycles.

#### **Lemma 4.2**

Consider a node with value $n$ ($n>b>a$) constructed such that the major and minor edges are attached to nodes with values $b$ and $a$, respectively. Then:

(i) If node $n$ is plain (black), any new node with major edge connected to $n$ has a minor edge connected to $n$’s minor parent $a$.
(ii) If node $n$ is flipped (orange), any new node with major edge connected to $n$ has a minor edge connected to $n$’s major parent $b$.

Thus consider Fig. 4b(iv), for example. Node $4$ extends from flipped node $2$ and so has major edge connected to $2$ and minor edge connected to $2$’s major node $1$. Node $5$ extends from plain node $4$ and so has major edge connected to $4$ and minor edge connected to $4$’s minor node, $1$.

This construction is termed the *2-d Poset Tree*, $P$, and will later be used to count the total number of evolutions in BFB space. However, we next use this construction to count the number of evolutions that correspond to a single fold sequence or word.

### Counting posets

We now have the 2-d tree poset to encapsulate the order relations between the fold reference positions, where a daughter node labeled $n>a,b$ has two parental nodes labeled $a$ and $b$ with corresponding fold reference positions that satisfy an inequality of the form $\min \{x_a,x_b\}<x_n<\max \{x_a,x_b\}$. In Fig. 4b(iv) this construction represents the inequalities $x_0<x_2<x_1$, $x_0<x_3<x_1$, $x_2<x_4<x_1$ and $x_4<x_5<x_1$. These inequalities are equivalent to two conditions; $x_0<x_3<x_1$ and $x_0<x_2<x_4<x_5<x_1$. That is, $x_3$ is free to roam between $x_0$ and $x_1$, and the order of $x_2$, $x_4$ and $x_5$ is fixed within the same range. We thus find that $x_2$, $x_4$ and $x_5$ split the region into four regions that $x_3$ can occupy; there are four possible orders.

Now if we just consider the major edges of the 2-d tree we get the tree structure of Fig. 4b(v). Note that we get two branches, one containing the node $3$, the other containing nodes $2$, $4$ and $5$. This is precisely how we just separated the four corresponding positions. We now find that nodes on the same branch have corresponding positions with a fixed order, whereas nodes on distinct branches correspond to positions for which no order relations exists. By ignoring the minor edges the 2d-tree construction then becomes a standard tree construction, which encapsulates the ordering information for the fold positions. This is termed the *Order tree*, $T$.

The number of possible orders for Fig. 4b(v) was found by counting the number of ways of intercalating the single position $x_3$ from one branch, with the three positions $x_2$, $x_4$ and $x_5$ on the other branch. This is the combinatorial term $4 \atopwithdelims ()1$, as in Fig. 4c(ii), and we would get similar terms at any branching nodes. This has a simpler representation. If we place $5!$ at the root node (the number of fold positions), and values $5,3,2,1,1$ at the remaining nodes (the sizes of maximal subtrees rooted at each node), the ratio $\frac{5!}{5\cdot 3\cdot 2\cdot 1\cdot 1}=4$ provides the correct count. We thus have the number of orders that correspond to fold sequence $[1,1,2,2,1]$ or word $1213145413121$. An explanation of these properties can be found in the appendix, where we prove the following result.

#### **Theorem 4.2**

Let $T$ denote the order tree of a 2-d poset tree $P$ deriving from a BFB word or fold sequence on $n$ BFB cycles. Tree $T$ then has $n+1$ nodes. If each node $b$ (except the root) has a label $m_b$ that is the size of the subtree rooted at that node, then the number of orders is given by $\Phi (T)=\frac{n!}{\prod _b m_b}$.

## The size of BFB space

We can now count the number of distinct evolutions for a single BFB word or fold sequence. Consider next two problems, firstly, how to count the number of different words given a fixed number of BFB cycles, and secondly how to then utilize Theorem 4.2 to count the total number of evolutions in BFB space.

We then first consider the question of how many different fold words can form from $n$ BFB cycles, both for the case of reduced and fold sequences. Although closed forms for these counts would seem intractable, we can derive counts recursively, where we have the following result.

### **Theorem 5.1**

Let $v_1=w_1=(1,0,0,\dots )$ denote the infinite vector with single unit entry. We construct general vectors $v_n$ with components $v_{n,m}$, where $m=1,2,\dots$ through the recursive relation $v_{n+1,m}=\sum _{k=\lfloor \frac{m+1}{2}\rfloor }^{m-1}v_{n,k}$. Then the number of reduced BFB sequences of length $n$ is $\sum _{m=1}^{\infty }v_{n,m}$. Applying the recursion $w_{n+1,m}=\sum _{k=\lfloor \frac{m+1}{2}\rfloor }^{\infty }w_{n,k}$ yields the number of full BFB sequences of length $n$ as $\sum _{m=1}^{\infty }w_{n,m}$.

The resulting counts can be seen in Table 1, where we see the number of sequences grow rapidly with BFB cycles. We now turn to the enumeration of distinct evolutions for each fold sequence, where we have the following result.

**Table 1:** Counts of distinct representative BFB sequences, evolutions and copy number profiles

BFBs	1	2	3	4	5	6	...	10
Reduced sequences	1	1	2	7	41	397		627,340,987
Reduced copy number profiles	1	1	3	19	247	6445		–
Reduced evolutions	1	1	3	21	315	9765		10,180,699,028,325
Full sequences	1	2	6	26	166	1626		2,290,267,226
Full copy number profiles	1	2	5	24	271	6716		–
Full evolutions	1	2	8	64	1024	32768		35,184,372,088,832

### **Theorem 5.2**

If $n$ BFB cycles take place then the total number of distinct evolutions is given by $2^{\frac{n(n-1)}{2}}$. The first BFB cycle $(n=1)$ produces a single fold. For $n \ge 2$, the total number of evolutions that retain at least one copy of all folds produced in the BFB process is given by $\prod _{i=1}^{n-1} (2^i-1)$. The proportion of evolutions that do not lose information then tends to limit $\prod _{i=1}^{\infty } (1-2^{-i})=0.288.$

We can also see these counts in Table 1, where we see the number of evolutions rising super-exponentially as a function of BFB count.

**Fig. 5:**
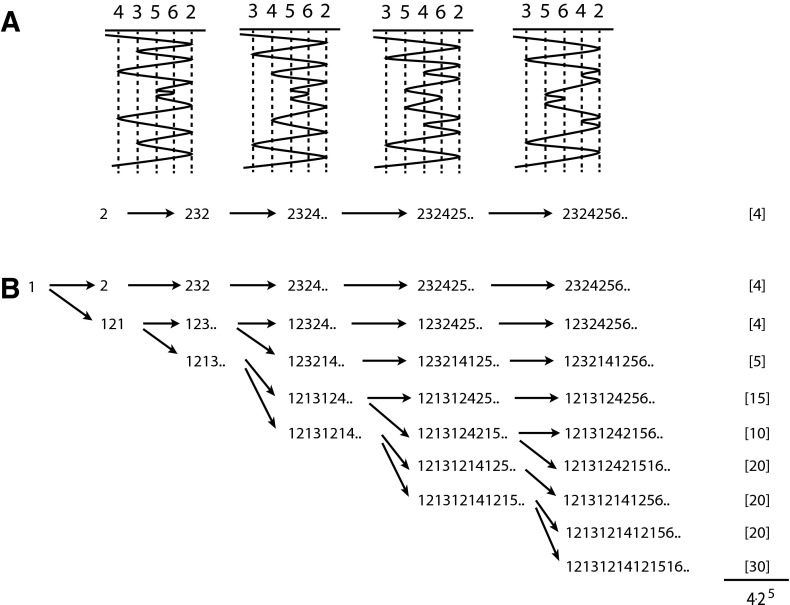
In **a** we have $4$ possible structures arising from the five fold word $2324256524232$. In **b** we see the introduction of a first fold gives rise to $4 \cdot 2^{5}$ possible structures

The proof of this theorem relies on an appropriate induction. This involves the reintroduction of a *first* fold. In Fig. 5a we see the four possible structures that correspond to the *five-fold* word $W=2324256524232$ based on five symbols $2,3,4,5,6$, along with the sequence of word operations that generate $W$. For each word $W=An\overline{A}$ in Fig. 5a, b we write $An..$ for brevity. In Fig. 5b we see several possible ways of introducing an initial fold $1$ that preserves the order of the other folds in the word. For example, from the word $1$ we can introduce fold $2$ before or after $1$ to give us $2$ or $121$. The word $2$ then follows the same evolution as Fig. 5a, whereas $121$ again provides two choices; remove the second $1$ to give $12321$, or introduce fold $3$ after the second copy of fold $1$ to give $1213121$. Note that both choices contain the original term $232$ as a subsequence. When we follow this decision process through all five folds we get nine words. We then calculate the number of orders for each word with Theorem 4.2 and find that we have $2^{5}$ times the original number of orders. To explain this we need to introduce a class of operations on the two-dimensional poset trees introduced above.

We constructed an order tree from the 2d-tree by removing all minor edges. We require the capacity to modify the shape of an order tree with the following operation.

**ES: edge switching**

Remove a major edge and insert the corresponding minor edge.

**Fig. 6:**
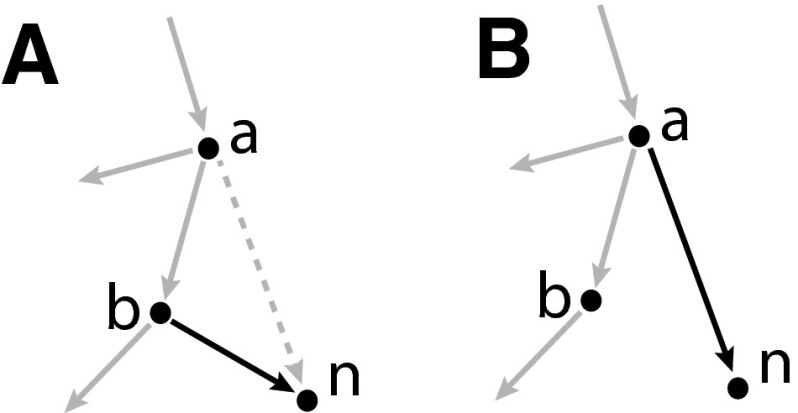
Edge switch operation: remove the major edge and insert the corresponding minor edge

This move can be seen in Fig. 6, and effectively moves a branch nearer to the root, and results in a tree structure. This move has no effect on the other edges or the nodes they are attached to. ES operations thus commute; we can perform the moves in any order and get the same structure.

This is a specific form of the Subtree Prune and Regraft (SPR) operation that has seen application to many other problems in evolution (Semple and Steel 2009).

We require a specific set of ES operations. Let $S(T)$ denote the set of connected subgraphs of a given order tree $T$ that contain the root node of $T$. $S(T)$ is therefore a set of subtrees of $T$. Then for any $s \in S(T)$, $T_s$ is the tree obtained from $T$ as follows.

**SS: subtree switching**

I Perform an ES operation on any flipped (orange) edge contained in subtree $s$.
II Perform an ES operation on any plain (black) edges adjacent to (so not contained in) subtree $s$.
III Form a new root node for $T_s$. Construct an edge from the new root to the root of $T$.

**Fig. 7:**
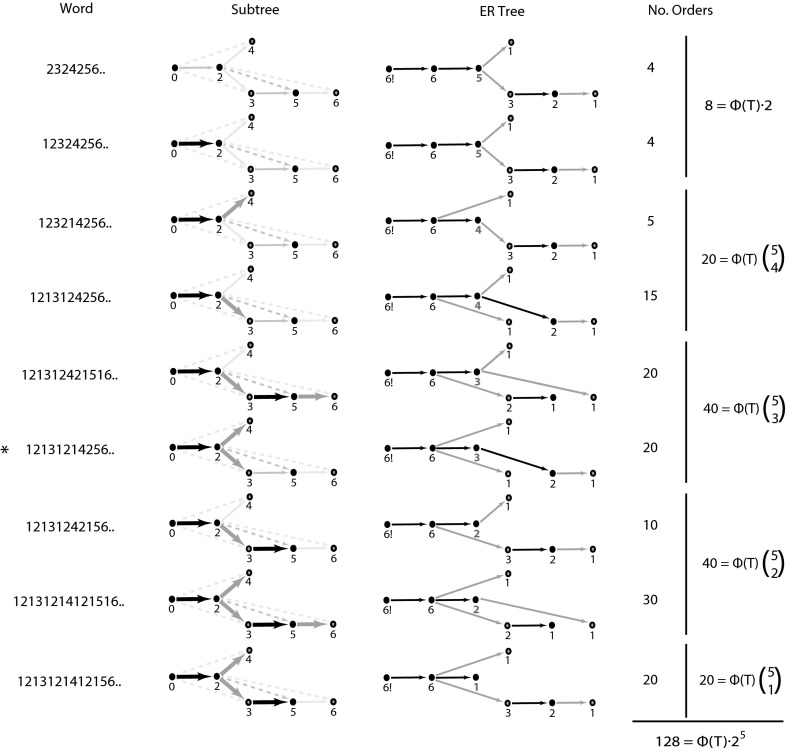
Subtree order counts are given for the tree corresponding to word 2324256524232 from Fig. 5. The *first column* indicates all nine words arising from introduction of fold $1$. The *second column* indicates corresponding 2-d tree subtrees in *bold*. The *third column* indicates the order tree after implementing SS operations. The *fourth column* counts the orders, where $\Phi (T)=4$ is the order count for the originating tree (see also Fig. 4c)

Examples can be seen in Fig. 7. In the row marked $*$ we see the subtree with the edges $02$, $23$ and $24$. Edges $23$ and $24$ are flipped, so we replace them with their corresponding minors (3rd column) (SS move **I**). Edge $02$ is untouched. Plain edge $35$ is adjacent to the subtree, so it is also switched (SS move **II**). We also have a new root node (SS move **III**).

We find there is a unique correspondence between the possible introductions of a first fold $1$ and the SS operations.

### **Lemma 5.1**

Let $T$ be the order tree with $n+1$ nodes associated with an $n$-fold word. Let $T'$ be the order tree with $n+2$ nodes associated with an $(n+1)$-fold word obtained by introducing a new first fold. Let $s$ denote the union of all paths from the root of $T$ to nodes $m$ such that the recursion $W_m=W_{m-1}(s_m-1)m\overline{W_{m-1}(s_m-1)}$ is replaced with $W_m=W_{m-1}(s_m-1)1m1\overline{W_{m-1}(s_m-1)}$ in the evolution of the word. Then $s$ is a subtree of $T$ and the order tree $T'$ is obtained by implementing SS operations on $s$.

This correspondence between the introduction of a new first fold and tree operations allows us to count a subset of order trees, as described below.

### **Lemma 5.2**

Let $T$ denote an order tree with $n+1$ nodes such that there is exactly one node $b$ directly below the root (so $n \ge 1$). For any subtree $s \in S(T)$ we let $b_s$ denote the number of descendant nodes from node $b$, plus one, after implementing SS on the subtree $s$. Then

$$\begin{aligned} \sum _{\{s \in S(T):b_s=r\}}\Phi (T_s)=\left\{ \begin{array}{ll} {n \atopwithdelims ()r}\Phi (T) &{}\quad r=1,2,\dots ,n-1 \\ 2\Phi (T) &{}\quad r=n \\ \end{array} \right. \end{aligned}$$

where $\Phi$ is the order function of Theorem 4.2.

Examples of this can be seen in Fig. 7, where the counts are broken down according to the values of node $b_s$ (in blue).

Summing over the possible values of $r$ then gives us $2^n\Phi (T)$.

### **Lemma 5.3**

For any order tree $T$ with $n+1$ nodes, $\sum _{s \in S(T)}\Phi (T_s)=\Phi (T)2^n$, where $T_s$ are defined by the Subtree Switching operations above.

An example of this can be seen in the last column of Fig. 7, where a graph corresponding to *five-fold* word $2324256524232$ and fold sequence $[1,1,2,2,1]$ with $4$ orders results in $4 \cdot 2^{5}$ new orders when a first fold is introduced.

We are finally in a position to prove our main result and count evolutions.

### *Proof of Theorem 5.2*

We have seen that any *n-fold* word with a corresponding order tree $T$ has $\Phi (T)$ possible structures. By Theorem 5.3, these are associated with $\Phi (T)2^n$ possible $n+1$-fold structures by the introduction of a new first fold. If we start with the trivial structure and inductively perform these fold introductions, we find that we have $1 \cdot 2^1 \cdot 2^2 \ldots 2^{n-1}=2^{1+2+\dots +n-1}$ possible evolutions. That is, there are $2^\frac{n(n-1)}{2}$ possible evolutions using $n$ folds, as required.

The formula counting evolutions that retain at least one copy of all folds is obtained as follows. The first BFB cycle results in a single fold retained in the structure. Consider any evolution on $i$ BFB cycles containing at least one copy of each fold produced. We then introduce a new first fold. We have seen there are $2^{i}$ ways to do this. All choices do not affect the number of copies of folds from BFB cycles $2,3,\dots ,i+1$. The only fold that we can lose all copies of is thus the first. There is then only one way to lose all copies of a fold, and that is for the first fold to be deleted in the second BFB cycle (such an example can be seen in the first line of Fig. 5b). The number of possible evolutions retaining copies of all $i$ folds is then $2^{i}-1$. For $n$ BFB cycles we multiply this factor across the $n-1$ introductions of a new first fold to give the desired expression. $\square$

The total number of distinct copy number profiles were determined for a fixed number of BFB cycles, as summarised in Table 1. Clearly the number of copy number profiles is smaller than the number of evolutions and there may be several evolutions for any given copy number profile. Furthermore, we can construct an infinite number of fold sequences with negative values that all reduce to any given reduced sequence. We thus need other methods to help identify the correct evolution for any given copy number profile.

So far we have treated BFB cycles as a discrete process, treating the folded structures as functions of fold sequences, a space we have now explored in some detail. However, the BFB process relies on the fold occurring somewhere along the length of the structure. We can thus consider the fold positions in a sequence of BFB structures as a stochastic process, and investigate the implications of this on the BFB structure.

## BFBs as a stochastic process

We first consider the stochastic nature of the structures length under the simplest assumption that the fold position is uniformly distributed along the structure. This is likely to be an approximation as rearrangements are known to be linked to fragility (Bignell et al. 2010), as well as transcription and chromatin conformation (Lemaitre et al. 2009). Selective pressure may also bias the uniformity of breakpoints. However, there is presently insufficient information to provide a precise distribution for BFB breakpoints along a genome, so in this section we establish what we can learn from the simplifying assumption of a uniform distribution. We will use this to show that the likelihoods for different fold sequences for each of $2^\frac{n(n-1)}{2}$ possible evolutions from $n$ BFB cycles can be markedly distinct.

### Length distributions

So far we have considered each BFB product as a structure folded with respect to a set of reference positions. We now imagine unfolding the entire structure at each stage.

**Fig. 8:**
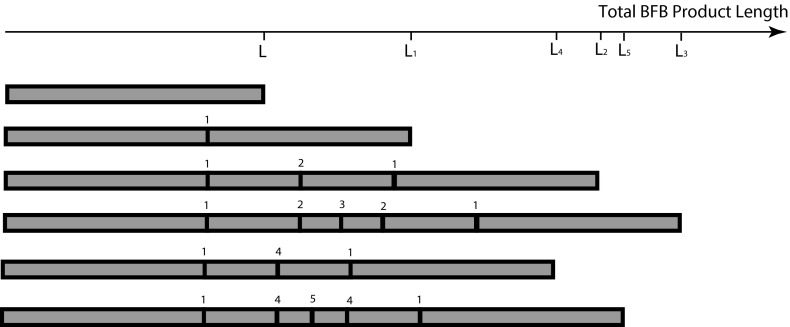
The lengths of the unfolded products of a sequence of five BFB cycles

Such an example can be seen in Fig. 8. Here we start with a product of length $L_0=L$. From Remark 3.1 we can assume that duplication is on the left side of the position of any BFB fold. The break occurs uniformly along this length, so after duplication, repair and unfolding, the next length $L_1 \sim U[0,2L_0]$. In Fig. 8, the first fold (position $1$) is beyond the midpoint of the previous structure (of length $L$), so the resulting structure increases in length, as it does for the next two BFB cycles. However, the fourth fold (position $4$) occurs in the first half of the previous structure, reducing the length and removing the second and third folds before the final BFB cycle again extends the structure.

We then see that the length $L_n$ is a stochastic Markovian process with conditionally uniform distribution $(L_n|L_{n-1}) \sim U([0,2L_{n-1}])$. The general length distribution $P(L_n)$ can then be derived, giving the following result.

#### **Theorem 6.1**

If $L_0=L$ is the initial length of the chord, then the length $L_n$ after the $n$th BFB cycle has distribution

$$\begin{aligned} P(L_n)=\left\{ \begin{array}{l@{\quad }l} \frac{1}{2^n (n-1)! L}log^{n-1}\left( \frac{2^{n+1}L}{L_n}\right) &{} L_n \le 2^nL \\ 0 &{} L_n>2^nL \\ \end{array} \right. \end{aligned}$$

with mean value $L$ and standard deviation $L\sqrt{(\frac{4}{3})^n-1}$.

Thus we find that although the lengths average value does not change, it is increasingly variable. We also see that the shortest distance $\frac{L_m}{2}$ of any copy of the $m$th fold from the ends is preserved. The first fold encountered is always a distance $\frac{L_1}{2}$ from either end, for example. All copies can be lost however, and we have seen in Fig. 8 that as the BFB process continues, BFB events that shorten the structure can delete all copies of folds from some previous BFB events. We can characterize these properties as follows.

#### **Theorem 6.2**

The original distance $\frac{L_m}{2}$ of the $m^\mathrm{th}$ BFB fold from the end of the structure is the shortest distance of any subsequent copy of that fold to either end. If $L_n<L_m$ and $n>m$ then all copies of the $m^\mathrm{th}$ BFB fold are permanently excised from the BFB product. Thus if the $m^\mathrm{th}$ BFB fold is to avoid extinction through a series of $N$ BFBs then $L_m<min_{\{n>m\}}L_n$. Subsequently, if we have a series $L_1,L_2,\dots ,L_N$ of BFB lengths, the only BFB folds that survive will be a subset with increasing length, in the same order that they occurred.

This raises two issues. Firstly, if we observe a sequence $L_1<L_2<\dots <L_N$ of BFB lengths in a final structure we would like to know how many folds from other BFB events have been completely excised from the genome in the process. Secondly, we know that the smallest length $L_1$ is the earliest remaining BFB. The fold at position $\frac{L_1}{2}$ is thus the first encountered as we traverse the structure. This also gives the position of the outermost fold relative to the reference. For example, in Fig. 4a(i), the first fold, at position $x_1$, is furthest from the ends of the structure, relative to the reference positions. The first fold thus measures the size of the amplicon.

A better understanding of the order statistics of the length sequence $L_n$ will help our understanding of both the scale of deleted BFB folds, and the size of amplicons, where we have the following result.

#### **Theorem 6.3**

The probability density $M_{k,N}(x,L)$ that the $k^\mathrm{th}$ BFB of a series $L_1,L_2,\dots ,L_N$ is the minimum with length $x$ is given by

$$\begin{aligned} M_{k,N}(x,L)=\frac{1}{2^{k}L}W_k(x,L)\left( 1-\sum _{i=1}^{N-k}\frac{1}{2^i}W_i(x,x)\right) \end{aligned}$$

where $L$ is the original length and,

$$\begin{aligned} W_k(x,y)=\int _x^{2y}\int _x^{2z_1}\dots \int _x^{2z_{k-1}}\frac{1}{z_1\dots z_k}dz_k\dots dz_1 = \sum _{j=0}^{k}a_{j+1}^k(x) \log ^j(2^ky), \end{aligned}$$

$a^k$ is the $k+1$ length vector $\prod _{r=1}^kB_r$, and $B_r$ is the $(r+1) \times r$ matrix

$$\begin{aligned} \left( \begin{array}{c c c c c} -\log (2^{r-1}x) &{} -\frac{1}{2}\log ^2(2^{r-1}x) &{} \cdots &{} -\frac{1}{r}\log ^r(2^{r-1}x) \\ 1 &{} 0 &{} \cdots &{} 0 \\ 0 &{} \frac{1}{2} &{} \cdots &{} 0\\ \vdots &{} \vdots &{} \ddots &{} \vdots \\ 0 &{} 0 &{} \cdots &{} \frac{1}{r}\\ \end{array} \right) \end{aligned}$$

We can use this to get the distribution of both the minimum length and its occurrence in the BFB sequence, as indicated in Corollary 6.1i–iii below.

The result also enables us to get the distribution of the amplicon size, that is, the position $L_{amp}$ of the outermost fold relative to the reference, $\frac{1}{2}min_{k \le N}\{L_k\}$, as summarized in Corollary 6.1iv.

We next consider an observed sequence of BFB folds with corresponding lengths $L_1<L_2<L_3<\dots <L_n$ and estimate how many BFBs were likely to have been deleted in this process. Specifically, if we have a sequence of BFBs with lengths $l_{1,1},l_{1,2},\dots ,l_{1,d_1},$ $L_1,l_{2,1},l_{2,2},\dots ,l_{2,d_2},L_2,\dots ,L_n$ such that $L_{i-1} \le L_i \le {l_{i,1},\dots ,l_{i,d_i}}$, then by Theorem 6.2 the BFB with length $L_i$ deletes the $d_i$ earlier BFB folds with longer lengths ${l_{i,1},\dots ,l_{i,d_i}}$ to leave the events $L_{i-1},L_i$. We can use Theorem 6.3 to estimate the scale of loss, $d_i$. If we have a BFB of length $L_{i-1}$, which is followed by the sequence $l_{i,1},\dots ,l_{i,d_i} \ge L_{i-1}$, then we require $l_{i,1},\dots ,l_{i,d_i}>L_i$, given that we start with length $L_{i-1}$. That is $Pr(l_{i,1},\dots ,l_{i,d_i} \ge L_i|L_{i-1}) =\frac{1}{2^dL_{i-1}}\int _{L_i}^{2L_{i-1}}\int _{L_i}^{2l_1}\dots \int _{L_i}^{2l_{d-1}}\frac{1}{l_1\dots l_{d-1}}dl_d\dots dl_1$. This can be calculated in much the same way as Theorem 6.3. A Bayesian inversion then allows us to estimate the distribution of $d_i$, given in Corollary 6.1v. In summary we have the following.

#### **Corollary 6.1**

(i) The probability that the $k$th of $N$ BFBs is the one with the minimum length $L_{min}$ is given by $\frac{M_{k,N}(L_{min},L)}{\sum _{k=1}^NM_{k,N}(L_{min},L)}$.
(ii) The probability density function of the minimum BFB length in a sequence of $N$ BFBs is given by $\sum _{k=1}^NM_{k,N}(L_{min},L)$.
(iii) The distribution of the number $N$ of BFBs for a given minimum length $L_{min}$ is then given by $Pr(N=n|L_{min})=\frac{\sum _{k=1}^nM_{k,n}(L_{min},L)}{\sum _{n=1} ^{\infty }\sum _{k=1}^nM_{k,n}(L_{min},L)}$.
(iv) The amplicon size, $L_{amp}$, given a sequence of $N$ BFB cycles has distribution $2\sum _{k=1}^NM_{k,N}(2L_{amp},L)$.
(v) If we observe two folds in a final BFB structure with consecutive lengths $L_{i-1}<L_i$ the number of BFBs occurring between them that are deleted by the $i$th BFB, $D_i$, has distribution, $Pr(D_i=d|L_{i-1}<L_i)=\frac{I_d}{\sum _{d=1}^{\infty }I_d}$, where $I_d=1-\frac{L_i}{2L_{i-1}}\sum _{k=0}^{d-1}\frac{1}{2^k} W_k(L_i,L_{i-1})$ and $W_0(L_i,L_{i-1})=1$.

**Fig. 9:**
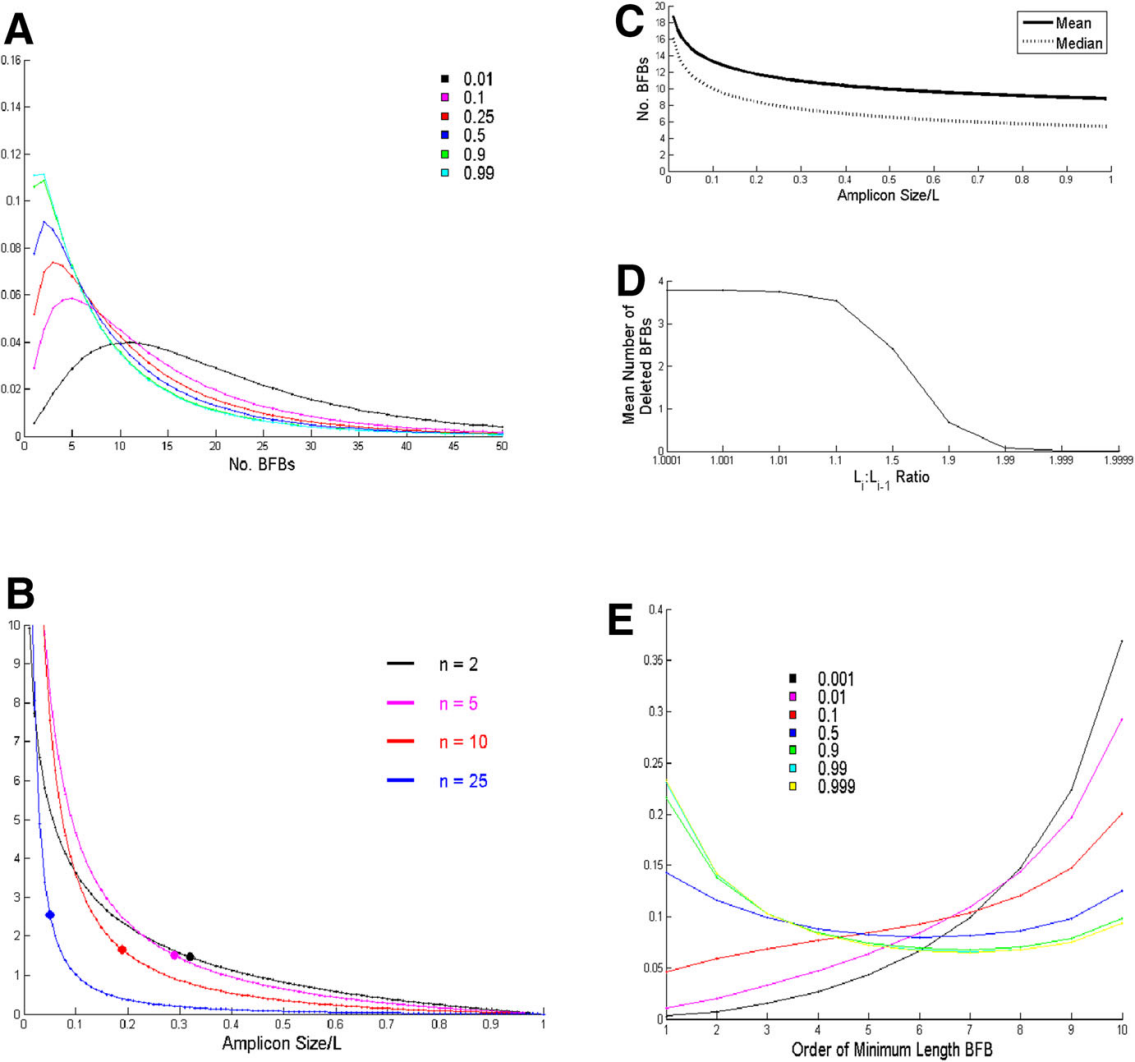
BFB distributions. **a** The distribution of BFB counts for a range of amplicon sizes (as a proportion of $L$). **b** The distribution of the amplicon size for a range of BFB counts. The mean positions are located at the *circles*. **c** The mean and median number of BFBs as a function of amplicon size. **d** The expected number of deleted BFB events as a function of the ratio $L_i:L_{i-1}$. **e** The distribution of the minimum length BFB for ten BFBs and a range of amplicon sizes

Some of these distributions are plotted in Fig. 9, where we see in b the trend that the outermost fold of the BFB, that is, the size of the amplicon, decreases as the number of BFB events increases.

This can be intuited as follows. If a BFB product has current minimum length $L_{min}$, then there is a chance the next fold will be smaller than $\frac{L_{min}}{2}$, deleting all previous folds, and reducing the position of the outermost fold. That is, the BFB process will result in atrophy of the amplicon size.

We also know from Theorem 6.1 that the average length of the structure does not change. This means that, on average, the same amount of DNA is present in a diminishing region of the reference genome, resulting in the localized high copy number structures typical of amplicons, such as Fig. 1c.

### Fold structure likelihoods

We have seen different BFB structures arising due to different fold sequences. It is thus natural to investigate the likelihood of a particular fold sequence occurring. We extend the stochastic process approach above to elucidate this problem. We again make the simplifying assumption that BFB folds occur uniformly along the length of the structure.

Suppose we are interested in the likelihood of observing fold sequence such as $\mathbf{r}=[1,1,2]$, the fourth structure in Fig. 3. We can build the likelihood inductively. The first fold $x_1$ is uniformly distributed across the original structure of length $L$, so we have $Pr(x_1|r_1)=\frac{1}{L}$. The second fold occurs at position $x_2$ on the second segment $(r_2=1)$ and so satisfies the inequality $x_0<x_2<x_1$. It is uniformly distributed along this segment, so $Pr(x_2|x_1,r_1,r_2)=\frac{1}{x_1-x_0}$. Now $Pr(r_3=2|x_1,x_2,r_1,r_2)$ is the chance of hitting the last of the four segments of the structure corresponding to $[r_1,r_2]=[1,1]$. This segment has length $x_1-x_0$ and the total length of the structure is $2(x_1-x_0)+2(x_1-x_2)=4x_1-2x_2-2x_0$. If we suppose $x_0=0$ and $L=1$ for simplicity then we get probability $\frac{x_1}{4x_1-2x_2}$. We can then can put this information together to get the probability of getting fold sequence $[1,1,2]$ conditional upon $[1,1]$ as follows:

$$\begin{aligned} P([1,1,2]|[1,1])&=\int _{0<x_2<x_1<1}P([1,1,2],x_1,x_2|[1,1])dx_1dx_2\\&=\int _{0<x_2<x_1<1}P([1,1,2]|x_1,x_2,[1,1])P(x_2|x_1,[1,1])P(x_1 |[1,1])dx_1dx_2\\&=\int _{0<x_2<x_1<1}P(r_3=2|x_1,x_2,r_1,r_2)P(x_2|x_1,r_1,r_2)P (x_1|r_1)dx_1dx_2\\&=\int _{0<x_2<x_1<1}\frac{x_1}{4x_1-2x_2}.\frac{1}{x_1}.\frac{1}{1}dx_1dx_2=\frac{1}{2}\log 2 \end{aligned}$$

This process can be applied in general which we summarized below.

#### **Lemma 6.1**

The likelihood of seeing reference positions ${x_n}$ for a given BFB sequence ${r_n}$ is given by, $P(x_1,x_2,\dots ,x_n|r_1,r_2,\dots ,r_n)=\prod _{i=1}^{n} \{ \frac{1}{x_{i_{max}}-x_{i_{min}}}\}$, where $x_{i_{min}}<x_i<x_{i_{max}}$ are the inequalities of the poset for ${r_n}$ given by Lemma 4.1.

The conditional probability of next fold sequence element $r_n$ is $Pr(r_n|x_1,\dots ,x_{n-1},$ $r_1,\dots ,r_{n-1})=\frac{x_{n_{max}}-x_{n_{min}}}{L_{n-1} (x_1,\dots ,x_{n-1})}$, where $L_{n-1}$ is the length after $n-1$ BFB cycles, a linear homogeneous function of $x_1,\dots ,x_{n-1}$. We then find

$$\begin{aligned} Pr(r_n|r_1,\dots ,r_{n-1})&=\int _{\Delta }Pr(r_n,x_1,\dots ,x_{n-1}|r_1,\dots ,r_{n-1})d\mathbf{x} \\&=\int _{\Delta }\frac{x_{n_{max}}-x_{n_{min}}}{L_{n-1} (x_1,\dots ,x_{n-1})}.\prod _{i=1}^{n} \left\{ \frac{1}{x_{i_{max}} -x_{i_{min}}}\right\} d\mathbf{x}, \end{aligned}$$

where $\Delta$ is the region defined by inequalities $x_{i_{min}}<x_i<x_{i_{max}}$.

**Fig. 10:**
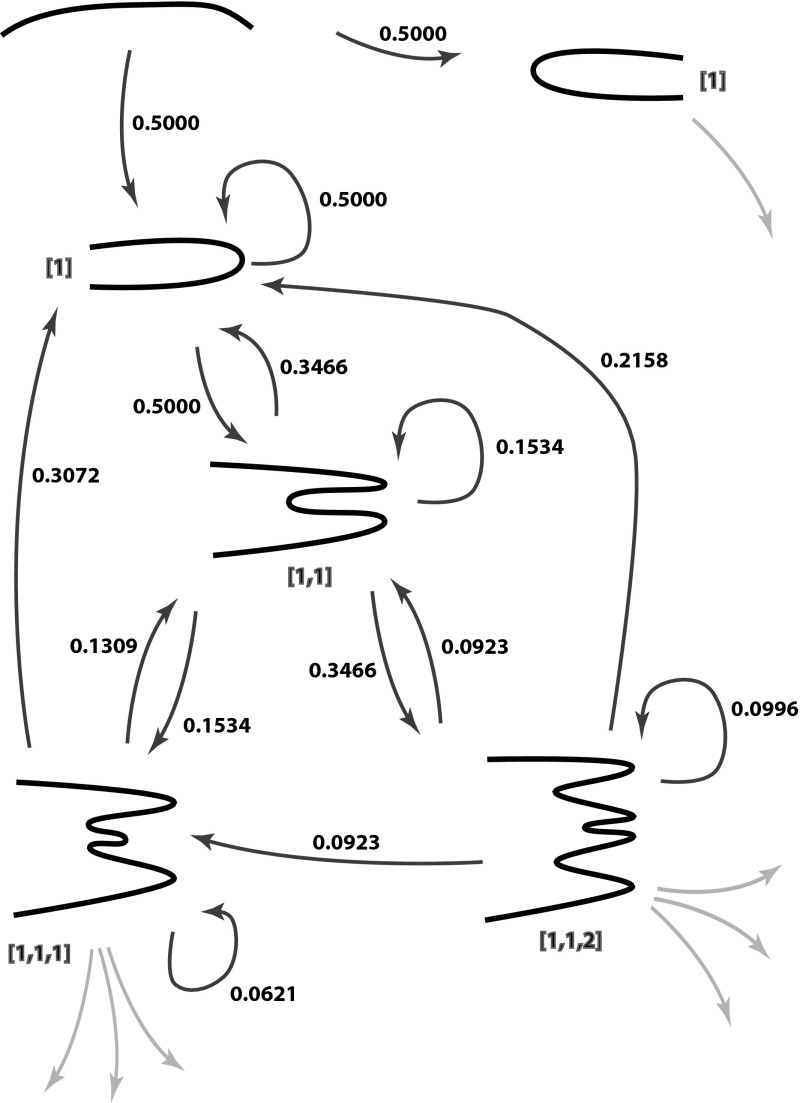
Probabilities of fold events in BFB space. The black chords indicate the structure for each BFB sequence (indicated in *red*). *Numbers alongside arrows* indicate the probability of taking that step (color figure online)

The probabilities for the first few BFB sequences can be seen in Fig. 10. These integrals rapidly become intractable and numerical methods are required. The simplest method is to randomly generate $x_1,x_2,..$ according to the conditional uniform distributions and average the simulated probabilities $P(r_n|x_1,x_2,\dots ,x_{n-1},r_1,\dots ,r_{n-1})$.

Note that these probabilities are not for reduced sequences. For example, although fold sequences $[1,1,1]$ and $[1,1,2,-1]$ reduce to the same structure, they take different paths through the evolutionary graph of Fig. 10 and have different likelihoods of occurrence. Multiplying the edge probabilities, we find the probabilities of arising are 0.038 and 0.008, respectively.

## Applications to amplicons in cancer genomes

We now put the framework we have described into context with some real data.

### Inference of BFB evolution

Consider the amplicon of Fig. 1c. This is a plot of paired end Illumina sequencing data from primary breast cancer sample PD4875, part of the collection for the International Cancer Genome Consortium (ICGC 2010). The amplicon lies in region 35–50 Mb of chromosome 11. Each black dot is a *read depth* measurement; a count of illumina reads lying within a $\approx$1 kb window. We used discordantly mapping reads to find rearrangement positions (Campbell et al. 2008). The red and green vertical lines are positions where events consistent with right and left facing BFB folds were detected, respectively. This resulted in six segmented regions $I$ to $VI$, separated by five breakpoints. Note that no segmentation method is necessary to determine the location of these breakpoints, the discordantly mapping reads have been used directly to reveal their location. Conversely, because we don’t use a segmentation method, we know the breakpoints, but not the copy number. Although no aberrantly mapping reads could be found at the junction between regions $I$ and $II$ (the green line is dotted to denote a putative BFB fold), this was likely due to mapping difficulties and the data are indicative of a structure formed by BFB cycles.

We thus have six regions, each with a fixed (unknown) copy number, and five folds. We would like to identify the underlying fold sequence and provide an explanatory evolution of events. This would indicate the order that the folds occurred in this process, the resultant structure, and subsequent copy numbers across the amplicon.

A natural approach would be to first determine integer copy numbers within each segment through a method of segmentation, and then explore the evolutionary space of BFBs to find a structure that fits the estimated copy number. This is an approach utilized in Greenman et al. (2011) for more general rearrangements.

The calculation of integer copy number remains challenging. There are several factors that can make this difficult, including variable mapability across the genome, the presence of subclonality, the presence of normal contamination, dispersion in read depth (Greenman et al. 2010; Klambauer et al. 2012; Nik-Zainal et al. 2012b; Loo et al. 2010; Xie and Tammi 2009). We circumvent these difficulties by estimating the rearrangement process and copy numbers simultaneously. To do this we make three simplifying assumptions. Firstly, we assume that the amplicon region we are considering contains a single BFB structure in a single clone, and there are no other clones (with observable signal) containing rearrangements in the region of interest. Secondly, we assume that the experimental signal (read depth) is a linear function of copy number. This is a realistic assumption that has been utilized in many other works (Alkan et al. 2009; Klambauer et al. 2012; Nik-Zainal et al. 2012b). Thirdly, we assume that we have identified all the breakpoints across the region of interest. Our approach is thus restricted to amplicons where the signal is clear and there is no missing data. We then proceed as follows.

An illumina sequencing library will produce a large number $N$ of reads (tens of millions). Each read will fall into a $\approx$1 kb bin with (small) probability $p=p(c)$, a linear function of the underlying integer copy number $c$ at each region. We then have a large value $N$ and small value $p$, so the read depth for each bin will be approximately Poisson distributed with rate parameter $Np$, where $p$ will be fixed across regions of constant copy number. Such an approach has been used in Xie and Tammi (2009), where a normal approximation to the distribution was also utilized. Differences in mapability across each bin result in dispersion and more precise distributions such as mixtures of Poissons may be necessary (Klambauer et al. 2012). For example, the mean read depths $z_i$ across all bins in each regions $i \in {I,II,III,IV,V,VI}$ of Fig. 1c were $\mathbf{z}=[154.7,519.8,398.2,465.2,305.5,186.3]$. The variances $\mathbf{\sigma ^2}=[986.0,6416.0,3981.6,4596.8,4830.3,2034.0]$ were notable larger. The individual read depths in region $i \in {I,II,III,IV,V,VI}$ will thus follow some distribution $\psi (\cdot )$ with mean value $\mu _i(c_i)$, linearly dependent upon the underlying copy number $c_i$, and standard deviation $\sigma _i$. The central limit theorem then tells us that $z_i$ will be well approximated by a normal distribution $N(\mu _i,\frac{\sigma _i^2}{m_i})$, where $m_i$ count the number of read depths in each region. For our example we had large values $\mathbf{m}=[5578,6716,3969,2536,8768,5366]$, so the approximation is reasonable.

We would now like to use the BFB machinery developed previously.

In order to analyse the amplicon, we first need to know whether the two ends of the BFB either both face left or both face right. The rightmost region, $VI$, has a higher signal than the leftmost, $I$. This is consistent with the ends of the BFB pointing in a rightward direction towards the centromere; region $I$ is not part of the BFB structure. This gives us five segmented regions, and so five folds to explain. The fold positions are labeled $x_j, j=1,\dots ,5$. We select the most likely evolution as follows.

For any proposed fold sequence $\mathbf{r} = [r_1,\dots ,r_n]$, we have a range of possible orders of breakpoints according to Theorem 4.2, each with an integer copy number profile $\mathbf{c} = [c_1,\dots ,c_n]$ that can be calculated. The mean read depth for region $i$ has normal distribution $(z_i|c_i) \sim N(\mu _i(c_i),\frac{\sigma _i^2}{m_i})$, where $\mu _i(c_i)=\alpha + \beta c_i$, and $\alpha$, $\beta$ are unknown parameters that represent the linear relationship between signal and integer copy number. The values $\sigma _i$ are calculated from the data and assumed fixed. We then construct likelihood $Pr(\mathbf{z},\mathbf{x}|\mathbf{r,c})=Pr(\mathbf{z}|\mathbf{x,r,c})Pr(\mathbf{x}|\mathbf{r,c})$. Now, the mean depths $z_i$ only depend upon the copy number profile, so $Pr(\mathbf{z}|\mathbf{x,r,c})=Pr(\mathbf{z}|\mathbf{x,c})=\prod _i{N(\mu _i(c_i),\frac{\sigma _i^2}{m_i})}$. The fold positions $Pr(\mathbf{x}|\mathbf{r,c})=Pr(\mathbf{x}|\mathbf{r})$ depend upon the fold sequence according to Lemma 6.1. This is maximized over $\alpha$ and $\beta$ and the likelihood recorded for any proposed BFB evolution.

The amplicon in Fig. 1c has five folds, and we obtained the likelihoods for 315 possible evolutions (see Table 1). The five highest values are listed in Table 2. The maximum likelihood solution suggests the actual copy numbers are $[16,12,14,6,2]$, corresponding to the fold sequence $[1,1,2,2,3]$. Note that the top two entries have identical copy number profiles, and so identical likelihoods $log Pr(\mathbf{z}|\mathbf{x,c})$, although the fold sequences are distinct.

**Table 2:** Top five likelihoods for evolution of Fig. 1c

Rank	BFB sequence	Copy number profile$^\mathrm{a}$	Order	$\log Pr(\mathbf{x}|\mathbf{r})$	$\log Pr(\mathbf{z}|\mathbf{x,c})$	Log-likelihood
1	[1,1,2,2,3]	[16,12,14,6,2]	[1,5,4,2,3]	$-$81.674	$-$921.1	$-$1002.774
2	[1,1,2,4,1]	[16,12,14,6,2]	[1,4,2,5,3]	$-$82.264	$-$921.1	$-$1003.364
3	[1,1,2,4,5]	[24,20,22,10,2]	[1,4,5,2,3]	$-$82.264	$-$2008.9	$-$2091.164
4	[1,1,2,2,1]	[12,8,10,6,2]	[1,5,2,4,3]	$-$81.674	$-$2056.7	$-$2138.374
5	[1,1,2,2,5]	[20,12,14,10,2]	[1,5,2,4,3]	$-$82.038	$-$2076.4	$-$2158.438

$^\mathrm{a}$ Copy numbers only include regions II–VI for chromosome undergoing BFB process, other chromosomes are ignored

The fold likelihood $Pr(\mathbf{x}|\mathbf{r})$ provides some hope to weakly distinguish these two cases, the more likely structure being given in Fig. 1d. In reality their ratio is insufficient to distinguish these two cases with statistical confidence. Furthermore, the fold likelihood also relies on assumptions that breakpoints are uniformly distributed along the length and should be viewed as approximate at best. However, even if we have a precise distribution for the breakpoint reference positions, it would seem probable that this component of the likelihood is going to have limited statistical power.

These unfortunate limitations will apply in general because evolutions do not always have unique copy number profiles (Table 1), and it is going to be difficult to uniquely identify the underlying evolution, even with good experimental data.

### BFB termination

**Fig. 11:**
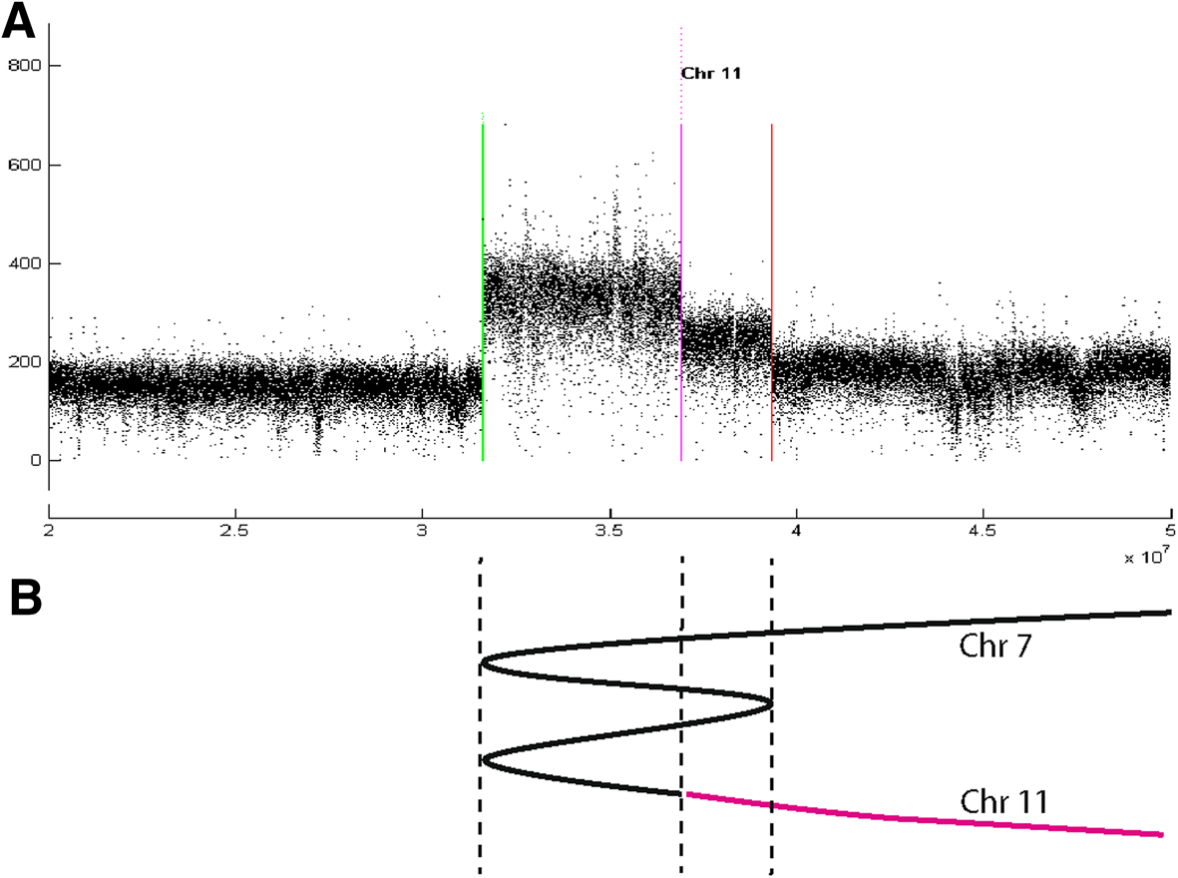
A BFB cluster in chromosome 7 of a primary breast cancer (PD4875). In **a** we see the amplicon; the two outer breakpoints are BFBs, the middle one a translocation to chromosome 11. **b** The genomic structure

The portrait of BFB cycles sketched in Fig. 1a would appear to continue indefinitely due to the continual production of chromosomes with two centromeres (dicentromy). However, this process will stop if we only have one centromere after DNA repair. This may be because a somatic telomere forms on one of the broken ends, but can also be because one of the exposed ends is attached to a different exposed end, on another chromosome for example, rather than the end of its duplication. This may result in a mono-centric chromosome, which will not break upon cell division, and the process can stop. This pattern can be observed in the data. In Fig. 11, for example, we see region 20–60 Mb on Chromosome 7 of a primary breast sample (PD4875) with three breakpoints, the two outermost of which are associated with BFB folds, and the middle one associated with a translocation to chromosome 11. This was likely to be the last step, terminating the breakage fusion bridge process, resulting in the structure portrayed in Fig. 11b.

### Clonality of BFB Process Under Selection

Our models have so far assumed that the BFB mutation process is randomly sampled and under no forces of selection. This is unlikely to be the case in general and the selection of any cells that have growth advantage are likely to emerge in cancer samples. We have seen in Fig. 1a that the BFB sequence arises due to spindles attached to the dicentromeric chromosome, dissecting the chromosome during cell division. This preserves the total amount of doubled DNA in both daughter cells. For a given break, we find the DNA is duplicated on one side of the break in one daughter cell, and on the other side in the other daughter cell. In Fig. 1a, for example, one daughter cell contains two yellow and four red genes, the other daughter cell two yellow genes, but the total number of red and yellow genes across both progeny is conserved at four. We then find that if the parent cell has fold sequence $[r_1,r_2,\dots ,r_{n-1}]$ with $s_{n-1}=\sum _{i=1}^{n-1}r_i$, and one daughter cell has sequence $[r_1,r_2,\dots ,r_{n-1},r]$, then the other daughter cell has sequence $[r_1,r_2,\dots ,r_{n-1},1-r]$. Given that we must have $-s_{n-1} < r \le s_{n-1}$, one of $r$ and $1-r$ must be non-positive.

If this process proceeds over $n$ cycles, and so $n$ cell divisions, producing $2^n$ cells, we are left to conclude that one of the cell lineages will consist entirely of positive terms and hence lose no BFB folds. This lineage is always gaining DNA by Theorem 4.1. Conversely, one of the lineages will consistently lose DNA. The $2^n$ cells will thus display a variety of copy number alterations, one of which may be advantageous to cancer. This cell may then emerge as a dominant clone.

We can also argue this from a different perspective. Note that the distribution of the amplicon size in Fig. 9b has a mean value that moves toward the origin as the number of BFBs increases. If a gene is, for the sake of argument, half way between centromere and telomere, and five BFB cycles take place, one can integrate this distribution up to that point to conclude that there is approximately a 95 % chance that the outermost fold is before the target gene and therefore only one copy of the gene is present (on the other allele) in the cell. Initially each cell has two copies of a gene. After 5 cell divisions there will be 32 cells and 64 copies of the gene target distributed amongst them. This implies that many copies of those genes are likely to be contained in one or two of those cells. Thus we find that it only takes relatively few BFB cycles to generate a cell containing multiple copies of a gene. If the gene is an oncogene, this cell then becomes a good target for selection and subsequent clonal expansion, producing the types of amplicons observed in cancer.

Selection thus plays a fundamental role in the evolution of these structures and a fuller investigation of selection acting across a growing set of cells undergoing a BFB process is warranted.

## Conclusions

We have highlighted some of the genomic complexities that arise from the BFB process that underlies the copy number profile of many amplicons observed in cancer. Although not every copy number profile can arise from a BFB process, the number of different BFB evolutions rises spectacularly quickly with the number of BFB cycles. Furthermore, a single copy number profile may be possible from more than one BFB evolution, making inference of the correct evolution difficult. For such degenerate cases, use of additional in-silico methods such as Greenman et al. (2011), or experimental methods such as Fluorescent In Situ Hybridisation (FISH), will be necessary to help identify the actual chromosomal structure and underlying process.

This work provides some understanding to the evolution of amplicons. However, amplicons can arise from other processes such as tandem duplication (McBride et al. 2012) or double minutes (Raphael and Pevzner 2004), for example, and amplicon evolution in general will be somewhat more complicated, possibly involving combinations of these processes, as well as other unexplored mechanisms.

This analysis also assumes that the data arise from a single dominant clone, which is not always the case (Nik-Zainal et al. 2012a, b). All of these other factors will have to be taken into account if we are to unravel more general evolutions of amplicons. However, the work presented is one step in that direction.

## Figures and Tables

**Fig. 12:**
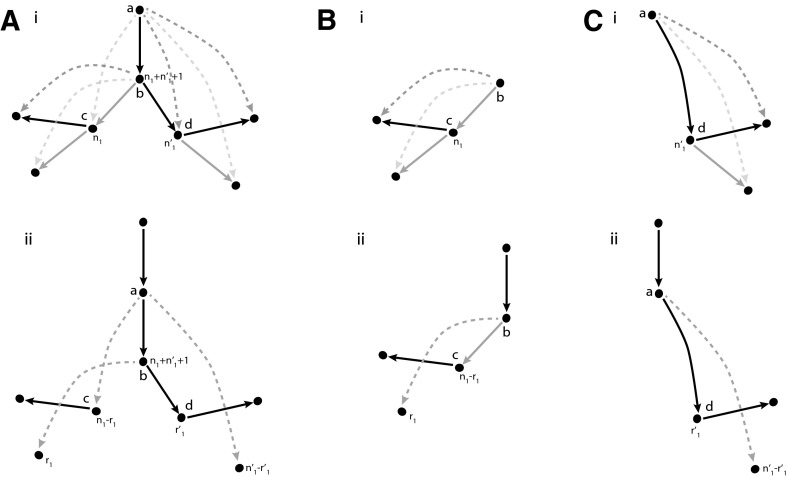
In **a**(*i*) we have a rooted tree with two branches. In **b**(*i*) and **c**(*i*) we have the two branches as distinct trees. In (*ii*) we have the corresponding trees after the SS operations. The node counts of the node $b$ below each root are indicated

## Appendix: Proofs

### *Proof of Theorem 3.1*

The final product of a BFB sequence must have a fold word of the form $Xn\overline{X}$ and so we can select the middle symbol, which must be unique as it is the last fold to form. Undoing this fold gives us the word $X$ to consider. Now $X$ is a product of a BFB, but fold $n$ may have truncated some sequence $Z$. $X$ must thus have the form $ZYm\overline{Y}$ for some subwords $Y$ and $Z$ (where an overline indicates symbols in reversed order). Now in any word generated by a BFB process, if we have two consecutive occurrences of a symbol $m_1$ then there must have been a BFB with fold $m_2$ that duplicated $m_1$ with fold $m_2$ between the two copies. Thus fold $m_2$ occurred later than $m_1$ and we have a word of the form $\dots m_1\dots m_2\dots m_1\dots$ . Note that the leftmost position of $m_1$ does not change position in the word when fold number $m_2$ is incorporated. The leftmost $m_2$ is to the right of the leftmost $m_1$ and we see that the first occurrences of fold symbols reflect their evolutionary order. If there is more than one symbol $m_2$ we can repeat the procedure, forming series $m_1,m_2,\dots$ until we find a symbol $m_n$ occurring once. This must exist because each fold symbol $m_i$ is located further into the word than $m_{i-1}$, and the word is finite in length. There may be more than one symbol occurring once (resulting in a word of the form $Xm_nm_{n+1}\dots m_{n+u}$ for unique symbols $m_n,\dots ,m_{n+u}$). The rightmost symbol $m_{n+u}$ must then be the latest event that we undo in STEP 2. Because the word is reduced in size at each step we either obtain a valid BFB evolution or the algorithm fails and the word is not a viable representation of a BFB process. $\square$

### *Proof of Theorem 4.1*

We show that $s_n$ counts positively labeled segments of the $n$th BFB structure inductively. This is true for $n=1$ because the structure after the first BFB cycle has two segments, and $s_1=r_1=1$ segment with positive label $1$. Assume true for $n=k$ so that we have $s_k>0$ positive values labeling the segments after the midpoint, and so $2s_k$ labels in total. Then we can select label $r_{k+1}$ for the next BFB with $-s_k<r_{k+1} \le s_k$. This means that if $r_{k+1}>0$ we duplicate $s_k$ non-positive and $r_{k+1}$ positive labels, producing $s_k+r_{k+1}=s_{k+1}>0$ new positive labels, as required. If $r_{k+1}<0$ then $s_k-(-r_{k+1})$ counts the number of (negative) labels that are duplicated, again producing $s_k+r_{k+1}=s_{k+1}>0$ new positive labels.

We then find that the structure formed by the $n$th BFB cycle has $2s_n$ segments, $s_n$ with positive labels and $s_n$ with non-positive labels, separated by the structures midpoint, which is the position of the fold formed by the $n$th BFB cycle.

Now, the $(k-1)$th and $k$th BFB folds occur on the $s_{k-1}$th and $s_k$th segment. Now if $r_k=s_k-s_{k-1}<0$, we have $s_{k-1}>s_k$. Now prior to the $k$th cycle we have $2s_{k-1}$ segments with the $(k-1)$th fold in the middle at the end of the $s_{k-1}$th segment. The $k$th cycle then duplicates the first $s_k(<s_{k-1})$ segments and deletes the rest, which contain the single copy of the $(k-1)$th fold. Thus if $r_k<0$, the $(k-1)$th fold is deleted.

Now consider the effect of $r_k<0$ on the fold sequence. After the $(k-2)$th event we have a structure that can be represented as a word $W_{k-2}$. The $(k-1)$th event duplicates the first $s_{k-1}$ segments, and so the first $s_{k-1}-1$ folds. Thus we can write $W_{k-2}=XY$ to find $W_{k-1}=X \cdot (k-1) \cdot \overline{X}$ for subwords $X$ and $Y$, where $X$ contains the first $s_{k-1}-1$ folds duplicated by the $(k-1)$th BFB cycle. The $k$th BFB cycle duplicates the first $s_k(<s_{k-1})$ segments, so $s_k-1(<s_{k-1}-1)$ folds, so we can write $X=UV$ to find $W_{k-1}=UV \cdot (k-1) \cdot \overline{UV}$ leads to $W_k=Uk\overline{U}$. However, we can instead write $W_{k-2}=XY=UVY$, skip the $(k-1)$th BFB cycle and duplicate the first $s_k-1$ folds. This results in the same word $W_k=Uk\overline{U}$. Thus, if $r_k<0$, we can drop the $(k-1)$th BFB cycle and get the same final structure. In terms of the cumulative sequence, instead of cumulative sequence $\mathbf{s}=[\dots ,s_{k-2},s_{k-1},s_k,\dots ]$, we delete the $s_{k-1}$ term to give, $\mathbf{s}'=[\dots ,s_{k-2},s_k,\dots ]$. To get the fold sequences we need the terms $r_k=s_k-s_{k-1}$. Then taking differences between consecutive terms in cumulative sequences $\mathbf{s}$ and $\mathbf{s}'$, the corresponding fold sequences $\mathbf{r}$ and $\mathbf{r}'$ are $\mathbf{r}=[\dots ,r_{k-2},r_{k-1},r_k,\dots ]$ and $\mathbf{r}'=[\dots ,r_{k-2},s_k-s_{k-2},\dots ]=[\dots ,r_{k-2},(s_k-s_{k-1}) +(s_{k-1}-s_{k-2}),\dots ]=[\dots ,r_{k-2},r_{k-1}+r_k,\dots ]$. Thus we find that if $r_k<0$, the two fold sequences $[\dots ,r_{k-1},r_k,\dots ]$ and $[\dots ,r_{k-1}+r_k,\dots ]$ produce identical final BFB structures, as required.

We have seen that the $k$th BFB structure contains $2s_k$th segments. The fold arising from the $k$th cycle is at the midpoint of the structure at the $s_k$th fold. The first fold of this structure points in direction $-1$, and the direction alternates with segments. We thus find that the $k$th fold points in direction $(-1)^{s_k+1}$, as required. $\square$

### *Proof of Lemma 4.1*

The fold word symbols indicate the order of folds occurring in the structure. From Theorem 4.1, if $r_n$ is the next value in the fold sequence, the segment this fold occurs on is $s_n$ from the end. This stretches between values $x_{W_{n-1}(s_n)}$ and $x_{W_{n-1}(s_n+1)}$. The value $s_n$ counts the segments from the start of the structure, which alternate in direction, so by Theorem 4.1, $d_n$ indicates which of $x_{W_{n-1}(s_n)},x_{W_{n-1}(s_n+1)}$ is larger, giving the inequalities specified. $\square$

### *Proof of Lemma 4.2*

We have two cases to consider. For (i), if node $n$ formed from $a$ and $b(>a)$ is plain, then we have word $..ab..$ becoming $..ana..$. When we then connect a new node $n'$ we have $..ana..$ becoming either $..an'a..$ or $..ann'na..$. In either case we construct a major edge from $n$ to $n'$, and a minor edge from $a$ to $n'$. For (ii), if node $n$ formed from $a$ and $b$ is flipped, then we have word $..ba..$ becoming $..bnb..$. When we then connect a new node $n'$ we have $..bnb..$ becoming either $..bn'b..$ or $..bnn'nb..$. In either case we construct a major edge from $n$ to $n'$, and a minor edge from $b$ to $n'$.

$$\square $$

### *Proof of Theorem 4.2*

We first establish that the reference positions corresponding to any chain of consecutive nodes in an order tree satisfy a single linear extension. We know from the construction of the 2-d poset tree for a fold word that any node corresponding to fold number $n>a,b$ has major and minor parental nodes with values $a$ and $b$. Prior to the formation of fold number $n$ we start with a segment that covers the interval $[x_a,x_b]$ in reference coordinates, where $x_a$ is the position of a left pointing fold and $x_b$ the position of a right pointing fold. The daughter node $n$ has a corresponding position $x_n$. If $n$ is a right (resp. left) pointing fold, we end up with a new segment $[x_a,x_n]$ (resp. $[x_n,x_b]$). In either case we end up with a new interval that is nested inside $[x_a,x_b]$. Any sequence of $I$ nodes $i_1,i_2,\dots ,i_I$ connected by major edges then correspond to a sequence of nested intervals. If $x_{j_1},\dots ,x_{j_J}$ are the corresponding positions for $J$ folds pointing to the left, and $x_{k_1},\dots ,x_{k_K}$ are the corresponding positions for $K$ folds pointing to the right (so $J+K=I$), they must then satisfy the single linear extension $x_{j_1}<x_{j_2}<\dots <x_{j_J}<x_{k_K}<\dots <x_{k_2}<x_{k_1}$, where $j_1<j_2,\dots <j_J$ and $k_1<k_2<\dots <k_K$. That is, any chain of consecutive nodes along a branch of the order tree correspond to one linear extension.

If we also have a second chain of $I'$ nodes that start from the same source node, but is otherwise distinct, then we have a second linear extension $x'_{j_1}<x'_{j_2}<\dots <x'_{J'}<x'_{K'}<\dots <x'_{k_2}<x'_{k_1}$ where either $x_{j_1}=x'_{j_1}$ or $x_{k_1}=x'_{k_1}$, due to the nested condition implying the earliest events are outermost. The total number of orders is then equal to the number of ways of intercalating $I-1$ and $I'-1$ positions (i.e. intercalate all positions except the common source node), which is the combinatorial term ${I-1+I'-1 \atopwithdelims ()I-1}$.

Now suppose more generally we have a node $r$ in an order tree with $B$ edges that descend from it, each directed towards $m_b$ descendant nodes, $b=1,2,\dots ,B$. Each set of $m_b$ nodes corresponds to $m_b$ reference positions. We suppose further that there are $z_b$ linear extensions for each set of $m_b$ nodes. We then find that if we take one linear extension from each of the $z_b$ possibilities, there are $\frac{m!}{\prod _{b=1}^{B}m_b!}$ ways of intercalating their positions, and so ${\frac{m!}{\prod _{b=1}^{B}m_b!}\prod _{b=1}^{B}z_b}$ linear extensions in total, where $m=\sum _{b=1}^Bm_b$. We thus find that if we associate $\frac{m!}{\prod _{b=1}^{B}m_b!}$ with node $r$, the total number of extensions is the product of these terms across all nodes in the order tree.

Now, the node $r$ is associated with term $\frac{m!}{\prod _{b=1}^{B}m_b!}$, and each of $B$ daughter nodes is similarly associated with a term of the form $\frac{(m_b-1)!}{\prod _{b=1}^{B'}m'_b}$. When multiplied together, the numerator of the daughter node, $(m_b-1)!$ cancels with the denominator $m_b!$ of the parent node to leave a term $m_b$ which we associate with the daughter node. The root node has no parents to cancel with and is then associated with numerator $n!$, where the order tree contains $n+1$ nodes in total. Multiplying these terms together then gives the desired result. $\square$

### *Proof of Theorem 5.1*

Now $v_{n,m}$ represents the number of reduced BFB sequences such that $s_n=\sum _{k=1}^nr_k=m$. $v_{1,m}$ is thus zero apart from the first unit entry. By the definition in Sect. 4.1 of reduced sequences, each sequence $[r_1,r_2,\dots ,r_n]$ can have a subsequent positive entry $r_{n+1} \in \{1,2,\dots ,m\}$. Thus, conversely, a sequence $[r_1,r_2,\dots ,r_{n+1}]$ of length $n+1$ and total $m$ must contain the sub-sequence $[r_1,r_2,\dots ,r_n]$ with a total ranging from $\lfloor \frac{m+1}{2}\rfloor$ to $m-1$. This gives us the relation $v_{n+1,m}=\sum _{k=\lfloor \frac{m+1}{2}\rfloor }^{m-1}v_{n,k}$. Summing $v_{m,n}$ over second index $m$ then counts the number of representative sequences of length $n$.

Similarly, $w_{n,m}$ represents the number of full representative sequences $[r_1,r_2,\dots ,r_n]$ such that $s_n=\sum _{k=1}^nr_k=m$. $w_{1,m}$ is thus zero apart from the first unit entry. By the definition in Sect. 4.1 of fold sequences, each such sequence can then have a subsequent entry $r_{n+1} \in \{-(m-1),-(m-2),\dots ,0,1,2,\dots ,m\}$. Thus, conversely, a sequence $r_1,r_2,\dots ,r_{n+1}$ of length $n+1$ and total $m$ must contain the sub-sequence $r_1,r_2,\dots ,r_n$ with a total ranging from $\lfloor \frac{m+1}{2}\rfloor$ to $\infty$. This gives us the relation $w_{n+1,m}=\sum _{k=\lfloor \frac{m+1}{2}\rfloor }^{\infty }w_{n,k}$. Summing $w_{n,m}$ over second index $m$ then counts the number of full representative sequences of length $n$. $\square$

### *Proof of Lemma 5.1*

We have three cases to consider.

For SS move I, suppose we have word $..ba..$ ($b>a$) becoming $..bmb..$. Then we have a minor flipped edge from $a$ to $m$ and major flipped edge from $b$ to $m$. To introduce first fold $1$ adjacent to $m$, we must start with a word of the form $..b1a..$. Node $b$ is adjacent to fold number $1$ and is part of subtree $s$. We then find that $..b1a..$ becomes either $..b1m1b..$ or $..bmb..$. From Lemma 4.2, in the former case we find node $m$ has a major edge connected to $a$, that is we have switched the major for minor edge and performed an ES operation. In the latter case we get the same result as before and no changes are made to major/minor status. Because $m$ is not adjacent to fold number $1$, edge $bm$ is not part of the subtree.

For SS move II, suppose we have word $..ab..$ ($b>a$) becoming $..ama..$. Then we have a minor plain edge from $a$ to $m$ and major plain edge from $b$ to $m$. To introduce first fold $1$ adjacent to $m$, we must start with a word of the form $..a1b..$. Node $b$ is adjacent to fold number $1$ and is part of the subtree. We then find that $..a1b..$ becomes either $..a1m1a..$ or $..ama..$. From Lemma 4.2, in the former case we find node $m$ has a major edge connected to $b$, that is we have the same major edge. Because fold $1$ is adjacent to $m$, edge $bm$ is in the subtree. In the latter case node $m$ has major edge connected to $a$, that is we have switched major for minor edge. Because $m$ is not adjacent to $1$, edge $bm$ is not in the subtree. That is, when the plain edge is adjacent to the subtree we switch, otherwise we leave alone.

For SS move III, suppose we have word $..a$ becoming $..ama..$. We have minor edge from $0$ to $m$ and major edge from $a$ to $m$. To introduce first fold adjacent to $m$, we start with a word of the form $..a1$. Node $a$ is adjacent to fold number $1$ and is part of the subtree. We then find that $..a1$ becomes either $..a1m1a..$ or $..ama..$. In the latter case $m$ still has major edge connected to $a$. In the former case $m$ now has major edge connected to $1$. If we replace the label for node $0$ with $1$, this is then equivalent to switching the major to the minor. We now have no node $0$, so we introduce a new node $0$, which is always connected to node $1$ during the first BFB event (SS move III). The case of word $a..$ becoming (single fold) word $m$ is similar.

The remaining edges have unmodified evolution and the order tree is unchanged.

These are all the moves required for SS operations on a subtree. $\square$

### *Proof of Lemma 5.2*

We prove the result by induction.

For the tree with two nodes $(n=1)$, we have two subtrees, both of which result in $b_s=1$ with an unchanged tree under SS operations, so $\sum _{\{s \in S(T):b_s=r\}}\Phi (T_s)=2\Phi (T)$ as required. We now assume the result is true for all such trees with $n$ nodes (or less) below the root. Now consider a tree with $n+1$ nodes below the root, such as Fig. 12a(i).

First consider the case $b_s = n+1$. There are two subtrees that leave the tree unchanged under SS operations. First is the empty subtree. The second subtree consists of the component of the tree composed of plain edges attached to the root. SS operations then leave the tree fixed and the order is the same. All other subtrees will send at least one edge to the root and reduce $b_s$. This gives us $\sum _{\{s \in S(T):b_s=n\}}=2\Phi (T)$.

Now consider the case that $b_s = r+1 < n+1$. There can be two types of edges descending from the node below the root (node labelled $b$ in Fig. 12). We can have a flipped edge, such as to node $c$, or a plain edge such as to node $d$. By Lemma 4.2, any children of flipped nodes $c$ must have minor edges connected to node $b$ below the root. No minor edges can touch the root. Conversely, any children of plain nodes such as $d$ must have minor edge attached to the root $a$, but not node $b$ below.

Suppose we have $i=1,2,\dots ,I$ indexing flipped nodes $c_i$ adjacent to $b$ and $j=1,2,\dots ,J$ indexing plain nodes $d_j$ adjacent to $b$. Any subtree $s$ of $T$ is going to result in a modified order tree such as Fig. 12a(ii) following SS operations. We restrict attention to subtrees that result in a tree $T_s$ such that node $b$ has value $b_s = r+1$. Prior to SS operations flipped node $c_i$ has value $n_i$. After SS operations this becomes $n_i-r_i$, for some value $r_i$, with $r_i$ nodes now descending from node $b$. Prior to SS operations plain node $d_j$ has value $n'_j$. After SS operations this becomes some value $r'_j$ with $n'_j-r'_j$ nodes now descending from $b$. If $\sum _ir_i+\sum _jr'_j=r$ node $b$ then has value $r+1$.

Now the subtree $s$ can be split into subtrees $s_i$ and $s'_j$ that pass through nodes $c_i$ and $d_j$. We also subdivide tree $T$ according to nodes $c_i$ and $d_j$ into $T_i$ and $T'_j$ as follows. For flipped nodes we take nodes $c_i$, their descendants, node $b$, and all minors attached to these nodes [such as Fig. 12b(i)]. For plain nodes we take nodes $d_j$, their descendants, node $a$, and all minors attached to these nodes [such as Fig. 12c(i)]. The subtrees $s_i$ and $s_j$ can then induce SS operations on corresponding trees $T_i$ and $T'_j$.

It is convenient to define ratios $R(T_s)=\frac{\Phi (T_s)}{\Phi (T)}=\frac{\prod _k m_k}{\prod _k m_{k,s}}$, where $m_k$ and $m_{k,s}$ denote the node values of node $k$, as described in Lemma 4.2, before and after the SS operations, respectively. Note that because the root node value (the total number of nodes in the original tree) is unaffected by SS operations, $m_{root}$ and $m_{root,s}$ cancel and do not contribute to $R(T_s)$.

We then find that,

$$\begin{aligned}&\sum _{\{s \in S(T):b_s=r+1\}}R(T_s) =\sum _{\{r_1+\dots +r_R=r\}}\sum _{\{s_1 \in S(T_{1,s_1}):b_s=n_1-r_1\}}\dots \sum _{\{s_I \in S(T_{I,s_I}):b_s=n_I-r_I\}}. \\&\quad \sum _{\{s'_1 \in S(T'_{1,s'_1}):b_s=r'_1\}}\dots \sum _{\{s'_J \in S(T'_{J,s'_J}):b_s=r'_J\}}\prod _{i=1}^I R(T_{i,s_i})\prod _{j=1}^J R(T'_{j,s'_j})\frac{1+\sum _i n_i+\sum _j n'_j}{1+r} \end{aligned}$$

where we get a product of terms $R(T_s)$ from each subtree, along with the last term corresponding to node $b$. Because trees $T_i$ and $T'_j$ have less than $n+1$ nodes we can use the inductive hypothesis and this sum becomes,

$$\begin{aligned}&\frac{1+\sum _i n_i + \sum _j n'_j}{1+r}\sum _{\{r_1+\dots +r_R=r\}}\sum _{\{s_1 \in S(T_1):b_s=n_1-r_1\}}R(T_{1,s_1})\dots \sum _{\{s_I \in S(T_I):b_s=n_I-r_I\}}R(T_{I,s_I}). \\&\quad \sum _{\{s'_1 \in S(T'_{1,s'_1}):b_s=r'_1\}}R(T_{1,s'_1})\dots \sum _{\{s'_J \in S(T'_{J,s'_J}):b_s=r'_1\}}R(T_{J,s'_J}) \\&\quad = \frac{1+n}{1+r}\sum _{\{r_1+\dots +r_R=r\}}\prod _{i=1}^I{^{n_i} C_{n_i-r_i}}\prod _{j=1}^J{^{n'_j}C_{r'_j}} = \frac{1+n}{1+r}\sum _{\{r_1+\dots +r_R=r\}}\prod _{i=1}^I{^{n_i} C_{r_i}}\prod _{j=1}^J{^{n'_j}C_{r'_j}} \\&\quad = \frac{1+n}{1+r}{^nC_r} = {^{n+1}C_{r+1}} \end{aligned}$$

Then substituting $R(T_s)=\frac{\Phi (T_s)}{\Phi (T)}$ gives the required result. $\square$

### *Proof of Theorem 6.1*

We abuse notation throughout and equate random variables with their values. The required result can be demonstrated with induction. The initial distribution ($n=1$) of $L_1$ is the uniform distribution $U([0,2L])$, reflecting the uniform choice of the first breakpoint in $[0,L]$ prior to duplication, in agreement with the formula for $P(L_1)$. At each step of the BFB process we pick a breakpoint from $U([0,L_{n-1}])$ and double the length of the retained piece. We thus have $P(L_n|L_{n-1})=\frac{1}{2L_{n-1}}, 0\le L_n \le 2L_{n-1}$. Then assuming the form for $n-1$ we find that, $P(L_n)= \int _{\frac{L_n}{2}}^{2^{n-1}L}P(L_n|L_{n-1})P(L_{n-1})dL_{n-1}$. An integration by parts then gives the desired form.

Now $(L_n|L_{n-1}) \sim U([0,2L_{n-1}])$ gives us the Martingale property that $E_{L_n|L_{n-1}}(L_n)=L_{n-1}$, thus the initial value $E(L_1)=L$ tells us that the mean length of the BFB segment is $L$.

The variance $\int L_n^2P(L_n)dL_{n}-L^2$ follows from an integration by parts. $\square$

### *Proof of Theorem 6.2*

This can be shown inductively. Initially, if the length of the structure produced by the $m$th BFB cycle is $L_m$, then clearly the fold at the midpoint is a distance $\frac{L_m}{2}$ from either end after the $m$th event. We then assume that all copies of the $m$th BFB fold are at least a distance $\frac{L_m}{2}$ from either end of the structure prior to the $n$th BFB (so $n>m$). One of two things can happen. Either the $n$th fold is nearer to the ends than $\frac{L_m}{2}$ (so $L_n<L_m$), in which case all the copies of the $m$th BFBs are deleted, or $L_n>L_m$ and some of them are duplicated, including a BFB nearest to the end, so the smallest distance $\frac{L_m}{2}$ is preserved, as required. $\square$

### *Proof of Theorem 6.3*

We first establish the formula for $W_k(x,y)$ by induction. We have initial value $W_1(x,y)=\int _x^{2y}\frac{dz}{z}=\log (2y)-\log (x)$ which matches the result. We assume true for $m$. Now we have $W_{m+1}(x,y)=\int _x^{2y}\frac{W_m(x,z)}{z}dz=\int _x^{2y} \sum _{j=0}^{m}a_{j+1}^{m}(x)\frac{\log ^j(2^{m}z)}{z}dz$. Integration gives $\int _x^{2y}\frac{\log ^j(2^{m}z)}{z}dz=\frac{1}{j+1}(\log ^{j+1} (2^{m+1}y)-\log ^{j+1}(2^{m}x))$ so that $W_{k+1}(x,y)=\sum _{j=0}^{m}a_{j+1}^{m}(x)\frac{1}{j+1}(\log ^{j+1} (2^{m+1}y)-\log ^{j+1}(2^{m}x))$. Thus we find $a_1^{m+1}(x)=-\sum _{j=0}^{m}\frac{1}{j+1}a_{j+1}^{m}(x)\log ^{j+1} (2^mx)$ and $a_{j+1}^{m+1}(x)=\frac{1}{j}a_{j}^{m}$, for $j \ge 1$, so that $a^{m+1}=B_{m+1}a^m$, as required.

Now if we have length $L_{n-1}$ prior to the $n$th BFB, then assuming the fold occurs uniformly along the length, the BFB duplication results in $P(L_n|L_{n-1})=\frac{1}{2L_{n-1}}$ with $0<L_n<2L_{n-1}$. The length sequence $L_n$ is also Markovian. Thus we can write $P(L_1,L_2,\dots ,L_n)=\prod _{k=1}^nP(L_k|L_{k-1})=\frac{1}{2^{n}L \prod _{k=1}^{n-1}L_k}$ where $L_n<2L_{n-1}<\dots <2^{n-1}L_1<2^nL_0=2^nL$.

The probability that the $k$th of $N$ BFBs have minimum length $L_k=x$ is given by $M_{k,N}(x,L)=Pr(L_1 \ge x,\dots ,L_{k-1} \ge x,L_k=x,L_{k+1} \ge x,\dots ,L_N \ge x)=Pr(L_1,L_2,\dots ,L_{k-1} \ge x,L_k=x)Pr(L_{k+1},\dots ,L_N \ge x|L_k=x)$, where we have used the Markovian property of the length sequence.

The first term can be obtained by integrating the above density,

$$\begin{aligned}&Pr(L_1,L_2,\dots ,L_{k-1} \ge x,L_k=x)\\&\quad =\frac{1}{2^{k}L}\int _x^{2L}\int _x^{2L_1}\dots \int _x^{2L_{k-2}} \frac{1}{L_1\dots L_{k-1}}dL_{k-1}\dots dL_1\\&\quad =\frac{1}{2^{k}L}W_k(x,L) \end{aligned}$$

The second term we similarly find as,

$$\begin{aligned}&Pr(L_{k+1}, L_{k+2},\dots ,L_N \ge x |L_k=x)\\&\quad =\frac{1}{2^{N-k}}\int _x^{2x}\int _x^{2L_k}\dots \int _x^ {2L_{N-1}}\frac{1}{L_k\dots L_{N-1}}dL_N\dots dL_{k+1}\\&\quad =\frac{1}{2^{N-k}}\int _x^{2x}\int _x^{2L_k}\dots \int _x^{2L_{N-2}} \left[ \frac{2}{L_k\dots L_{N-2}}-\frac{1}{L_{k+1}\dots L_{N-1}}\right] dL_{N-1}\dots dL_{k+1} \\&\quad =\frac{1}{2^{N-k-1}}\int _x^{2x}\int _x^{2L_k}\dots \int _x^{2L_{N-2}} \frac{1}{L_k\dots L_{N-2}}dL_{N-1}\dots dL_{k+1}-\frac{1}{2^{N-k}} W_{N-k}(x,x) \\&\quad \vdots \\&\quad =1-\sum _{i=1}^{N-k}\frac{1}{2^i}W_i(x,x) \end{aligned}$$

Putting these two terms together gives the required form. $\square$

### *Proof of Corollary 6.1v*

The required integral can be recursively split as follows:

$$\begin{aligned}&\frac{1}{2^dL_{i-1}} \int _{L_i}^{2L_{i-1}}\int _{L_i}^{2l_1}\dots \int _{L_i}^{2l_{d-1}}\frac{1}{l_1\dots l_{d-1}}dl_d\dots dl_1 \\&\quad =\frac{1}{2^dL_{i-1}}\int _{L_i}^{2L_{i-1}}\int _{L_i}^{2l_1}\dots \int _{L_i}^{2l_{d-2}}\left[ \frac{2}{l_1\dots l_{d-2}}-\frac{L_i}{l_1\dots l_{d-1}}\right] dl_{d-1}\dots dl_1 \\&\quad =\frac{1}{2^{d-1}L_{i-1}}\int _{L_i}^{2L_{i-1}}\int _{L_i}^{2l_1}\dots \int _{L_i}^{2l_{d-2}}\frac{1}{l_1\dots l_{d-2}}dl_{d-1}\dots dl_1- \frac{L_i}{2^{d}L_{i-1}}W_{d-1}(L_i,L_{i-1}) \\&\quad \vdots \\&\quad =1-\frac{L_i}{2L_{i-1}}\sum _{k=0}^{d-1} \frac{1}{2^k}W_k(L_i,L_{i-1}) \\ \end{aligned}$$

$$\square $$
